# The Therapeutic Potential of Farm Dust Extracts in a Mouse Model of Eosinophilic Inflammation

**DOI:** 10.1111/all.70121

**Published:** 2025-10-22

**Authors:** Rabia Ülkü Korkmaz, Jimmy Omony, Xiaomei Tan, Markus Klotz, Guilherme Dragunas, Sirui Chen, Soni Shankhwar, Zeynep Ertüz, Christoph Müller, Mohab Ragab, Aicha Jeridi, Romina Augustin, Janna Nawroth, Theodore S. Kapellos, Bettina Rankl, Ali Önder Yildirim, Erika von Mutius

**Affiliations:** ^1^ Helmholtz Zentrum München, Research Center for Environmental Health Institute of Asthma and Allergy Prevention Neuherberg Germany; ^2^ Comprehensive Pneumology Center (CPC‐M) Institute of Lung Health and Immunity (LHI), Helmholtz Munich Munich Germany; ^3^ Department of Pharmacy—Center for Drug Research Ludwig‐Maximilians Universität München Munich Germany; ^4^ Department of Internal Medicine II, TUM University Hospital, Klinikum Rechts der Isar, TUM School of Medicine and Health Technical University of Munich Munich Germany; ^5^ German Center for Lung Research (DZL) Munich Germany; ^6^ Helmholtz Pioneer Campus Helmholtz Zentrum München Neuherberg Germany; ^7^ Institute of Biological and Medical Imaging Bioengineering Center, Helmholtz Zentrum München Neuherberg Germany; ^8^ Chair of Biological Imaging at the Central Institute for Translational Cancer Research (TranslaTUM), School of Medicine and Health Technical University of Munich Munich Germany; ^9^ Faculty of Engineering Sciences University of Heidelberg Heidelberg Germany; ^10^ Institute of Experimental Pneumology, LMU University Hospital Ludwig‐Maximilian's University Munich Germany; ^11^ Ludwig‐Maximilians University Dr. von Hauner Children's Hospital Munich Germany

**Keywords:** asthma, epithelial barrier, farm dust extract, prophylactic intervention, regulatory T cells, Th2 inflammation

## Abstract

**Background:**

Asthma affects over 355 million people globally and poses a major healthcare burden. While corticosteroids remain a cornerstone of treatment, their side effects highlight the need for additional therapeutic strategies. Environmental exposures such as traditional farm dust have been linked to protection against asthma and allergies. This study investigated the therapeutic potential of farm dust extract (FDE) in a murine model of allergic asthma when administered after sensitization and during allergen challenge, mimicking a secondary prevention or early interventional treatment approach.

**Methods:**

We used an ovalbumin (OVA)‐induced asthma model to evaluate FDE effects on airway eosinophilia, airway hyperresponsiveness (AHR), mucus production, and IgE levels. Mechanistic studies assessed regulatory T cells (Tregs), dendritic cell phenotype, epithelial barrier integrity, and cytokine signaling. Complementary experiments were performed in peripheral blood mononuclear cells (PBMCs) from asthmatic donors.

**Results:**

FDE significantly reduced airway inflammation and AHR, with secondary prevention effects comparable to systemic dexamethasone. FDE enhanced Treg frequency and CTLA‐4 expression, modulated dendritic cell MHC‐II and PD‐L1 expression, and promoted an immunoregulatory environment. It also restored epithelial barrier integrity and increased IL‐33 release, supporting Treg activation. In asthmatic PBMCs, FDE increased Tregs, reduced Th2 cells, and suppressed *CIITA*, suggesting similar immune‐regulatory effects. Interactions among IL‐33, amphiregulin (AREG), and Tregs highlighted a mechanism reinforcing immune‐epithelial homeostasis.

**Conclusion:**

FDE administered after sensitization and during allergen challenge mitigated key asthma features in mice and showed translational potential in human cells, supporting its development as a novel, environmentally derived immunomodulatory strategy.

## Graphical abstract

Farm dust extract (FDE) treatment reduces airway eosinophilia, mucus overproduction, and MHC‐II expression on DCs, limiting antigen presentation and Th2 inflammation. It increases PD‐L1 on DCs, promoting T cell deactivation. Additionally, FDE enhances Tregs and upregulates CTLA‐4, reinforcing suppression. These effects mitigate airway hyperresponsiveness, highlighting FDE's potential for asthma therapy. AREG, amphiregulin; CIITA, MHC class II transactivator; CTLA‐4, cytotoxic T‐lymphocyte‐associated protein 4; DC, dendritic cells; EOS, eosinophil; FDE, farm dust extract; IgE, immunoglobulin; IL, interleukin; MHC‐II, major histocompatibility complex II; OVA, ovalbumin; PBMCs, peripheral blood mononuclear cells; PD‐L1, programmed death‐ligand 1; TGF‐β, transforming growth factor beta; Th2, T helper 2 cell; Treg, regulatory T cell.

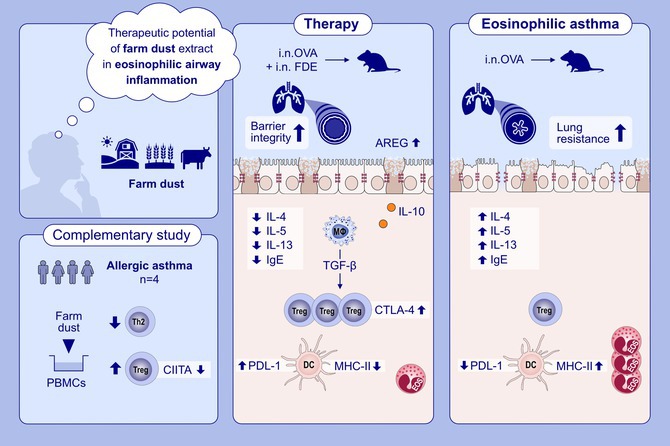

**Abbreviations**
AREG: amphiregulin
CIITA: MHC class II transactivator
CTLA‐4: cytotoxic T‐lymphocyte‐associated protein 4
DC: dendritic cells
EOS: eosinophil
FDE: farm dust extract
IgE: immunoglobulin
IL: interleukin
MHC‐II: major histocompatibility complex II
OVA: ovalbumin
PBMCs: peripheral blood mononuclear cells
PD‐L1: programmed death‐ligand 1
TGF‐β: transforming growth factor beta
Th2: T helper 2 cell
Treg: regulatory T cell

## 1 Introduction

Asthma is a chronic respiratory condition resulting in a significant individual and societal burden because of patient suffering, reduced quality of life, and increased healthcare costs [1]. Recent research and drug development in asthma have focused on understanding the disease's underlying mechanisms to improve treatment options.

Inhaled corticosteroids (ICS) are the cornerstone of asthma management and have significantly reduced asthma morbidity and mortality by controlling airway inflammation, improving lung function, and preventing exacerbations [2, 3, 4, 5, 6, 7]. In contrast, systemic corticosteroids are typically reserved for short‐term use during acute exacerbations or in severe, treatment‐resistant cases. Despite these limitations, dexamethasone is commonly used in preclinical models as a representative systemic corticosteroid due to its potency, stability, and well‐characterized anti‐inflammatory profile [2, 8]. In our study, dexamethasone was therefore employed as an experimental reference to evaluate the relative anti‐inflammatory efficacy of the tested intervention. Given the long‐term side effects associated with systemic corticosteroids—including immune suppression, osteoporosis, hypertension, and increased infection risk—even with short‐term use, there remains a critical need to identify safer and more targeted therapeutic alternatives for asthma management [8].

Ideally, alternative medicines should target the main features of asthma, that are, airway inflammation, airway eosinophilia, airway hyperresponsiveness, and epithelial barrier disruption. Given that airway eosinophilia is a hallmark of eosinophilic asthma—a common but distinct asthma subphenotype—our approach primarily addresses this phenotype. There is very robust evidence that asthma and allergies can be prevented by growing up on traditional farms [9, 10]. These observational studies have been corroborated by experimental work confirming the asthma–allergy preventive effect of extracts from environmental samples collected on farms [11, 12, 13, 14, 15]. It remains, however, unclear whether such exposures also have therapeutic potential, which so far can only be tested experimentally.

## 2 Results

### 2.1 Exposure to Farm Dust Extracts Improves Airway Function and Reduces Cell Recruitment in OVA‐Induced Experimental Asthma

**FIGURE 1:**
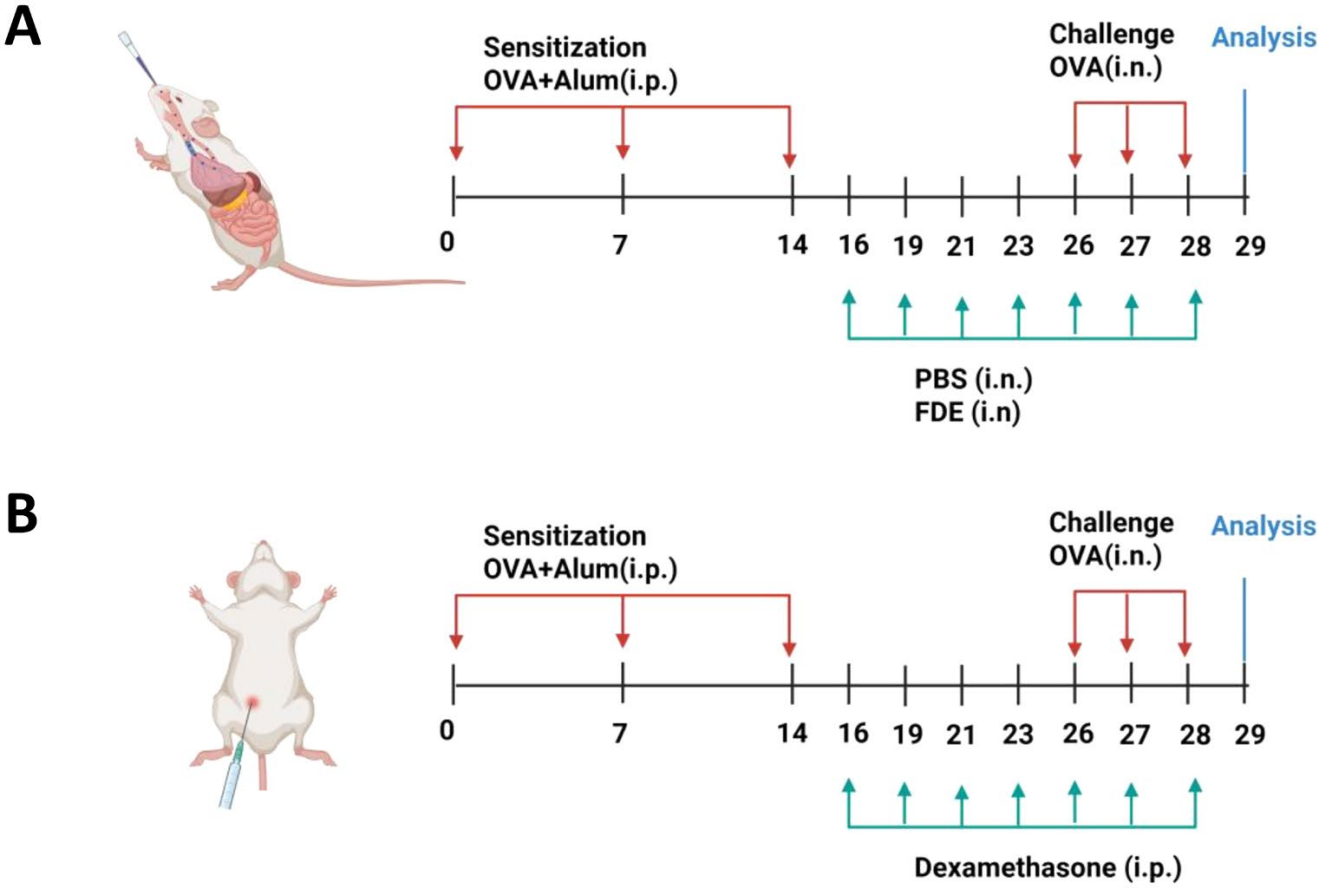
Experimental ovalbumin (OVA)‐driven allergic asthma model. (A) Briefly, mice were immunized by intraperitoneal (i.p.) injection with OVA‐Alum (50 μg/1 mg) on days 0, 7, and 14, challenged intranasally (i.n.) with 100 μg OVA (in 25 μL PBS) on days 26, 27, and 28. FDE. As a control, mice were sensitized with PBS on days 0, 7, and 14, and were intranasally administered 32 mg/mL (in 25 μL PBS) on days 16, 19, 21, 23, 26, 27, and 28. The mice were sacrificed, and organs were harvested 24 h after the last administration for single‐cell analysis. (B) Experimental OVA‐driven allergic asthma treatment model. In this model, in addition to the protocol in this figure, dexamethasone (0.5 mg/kg) was administered intraperitoneally on days 16, 19, 21, 23, 26, 27, and 28. Airway hyperresponsiveness (AHR) was measured, and tissue samples were collected for further analysis.

Previous studies investigating the protective farm effect on experimental allergic asthma were initially shown in an OVA‐induced allergic asthma mouse model [14]. To explore whether exposure to farm dust extract (FDE) might also serve as secondary prevention of asthma, which may equal an early therapeutic intervention, FDE was administered following sensitization and during the OVA challenge phase to mimic an early treatment strategy and included the corticosteroid dexamethasone as a control (Figure 1A,B).

**FIGURE 2:**
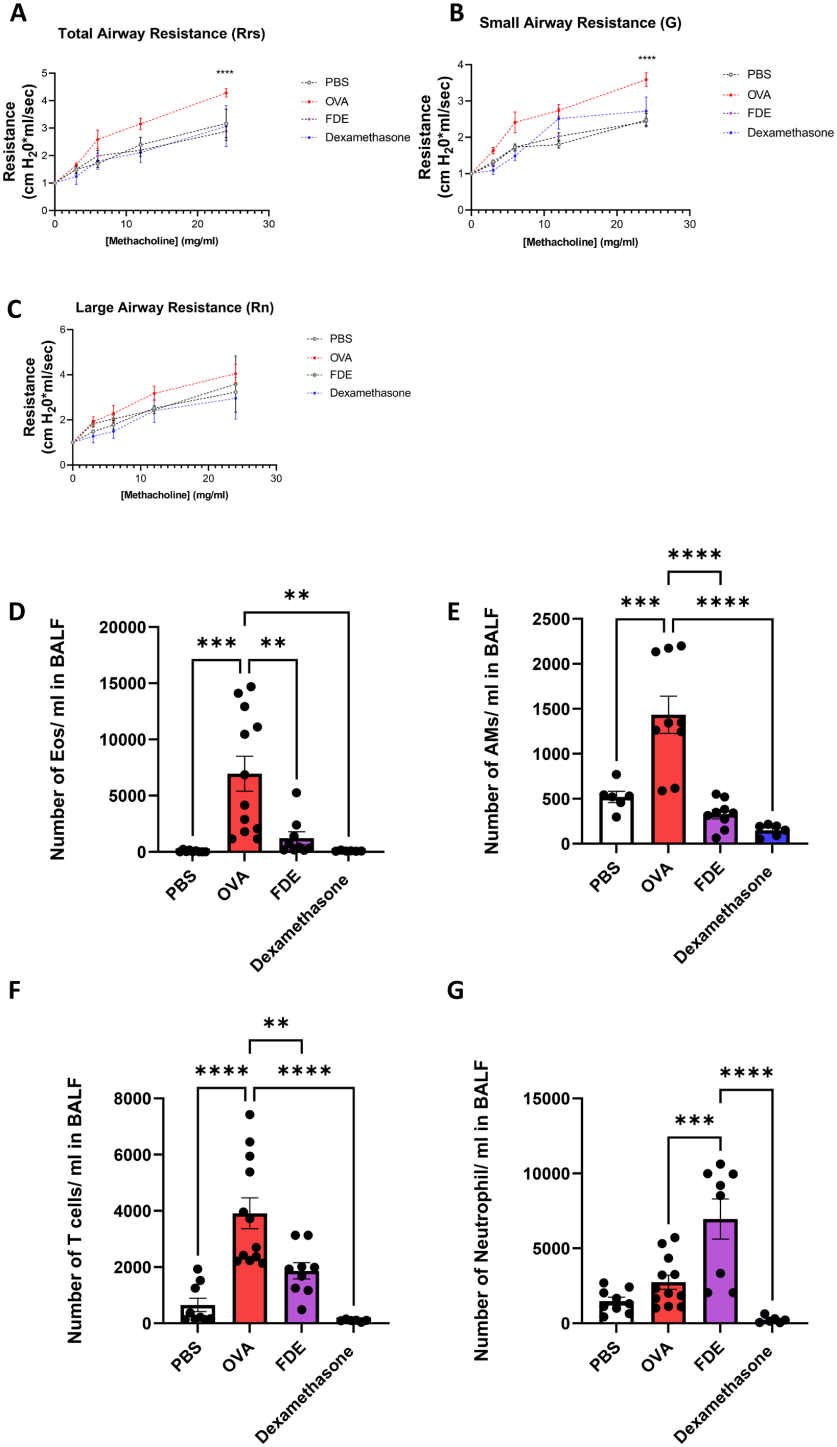
FDE orchestrates airway hyperresponsiveness and immune cells. AHR determined after i.n, PBS‐ (dashed white), OVA‐challenged (dashed red), and FDE treatment (dashed purple), or i.p. dexamethasone treatment (dashed blue). AHR was measured as airway resistance in response to methacholine exposure. (A) Total airway resistance (Rrs), (B) small airway resistance (G), (C) large airway resistance (Rn) values shown are the mean ± SEM; *n* = 6–15 per group. Data were analyzed using two‐way ANOVA followed by a Bonferroni post hoc test. * shows significant differences between OVA‐ and FDE‐treated groups, **p < 0.01, ***p < 0.001, ****p < 0.0001, (D) quantification of eosinophil numbers in bronchoalveolar, (E) quantification of alveolar macrophage number (AMs) in BALF, (F) quantification of neutrophil number in BALF, (G) quantification of T cells number in BALF, fluid (BALF) (PBS—white bar), OVA‐challenged (red bar), and FDE (purple bar), or i.p. dexamethasone treatment (blue bar). Data were analyzed by ANOVA followed by Tukey's post hoc test.

The Forced Oscillation Techniques (FOT) were used to assess total resistance of the respiratory system (RRS), Newtonian resistance (resistance attributable to large airways [RN]), and tissue damping (resistance attributable to small airways; G). FDE significantly reduced airway hyperresponsiveness (AHR), as indicated by a marked decrease in total and small airway resistance (Figure 2A,B), while no significant change was observed in large airway resistance (Figure 2C). Notably, FDE, like dexamethasone, reduced AHR levels comparable to the PBS control, suggesting that FDE mimics the therapeutic effects of dexamethasone in this model. Furthermore, lung compliance (Crs) and lung elastance (Ers) remained unchanged, suggesting that FDE did not impair lung mechanics and thus did not induce mechanical stiffness (Figure S1A,B). Histological analysis of H&E‐stained lung sections revealed pronounced inflammatory cell infiltration in OVA‐treated mice, accompanied by epithelial thickening and airway wall remodeling. In contrast, mice treated with FDE or dexamethasone showed markedly reduced inflammatory cell infiltration with preserved lung architecture (Figure S5B). To ensure that repeated FDE administration does not promote fibrotic or COPD‐like tissue remodeling, we examined key gene signatures associated with fibrosis and COPD using single‐cell transcriptomic analysis across epithelial and immune cell populations (Figure S6A–D). We did not observe an upregulation of these markers in FDE‐treated mice. These findings suggest that short‐term, repeated intranasal FDE exposure does not elicit fibrotic or COPD‐like responses at the molecular or tissue level.

To characterize the immune cell populations recruited to the airways following OVA exposure, bronchoalveolar lavage fluid (BALF) was collected, and cells were analyzed using flow cytometry. As expected, OVA exposure significantly recruited macrophages, eosinophils, and T cells into the BALF (Figure 2D–F). Therapeutic application of FDE significantly reduced eosinophil and macrophage numbers following the OVA challenge. In turn, FDE treatment elevated the number of neutrophils in the BALF of these mice (Figure 2G). We performed single‐cell analysis to assess the impact of FDE treatment on neutrophil phenotype in the lung, focusing on key surface markers, including *Cd11b* (Itgam), *Cd101*, *Cd11c*, *Cxcr2*, and *Cxcr4*. A heat map revealed significant changes in neutrophil profiles: *Cd11b* and *Cd101*, associated with adhesion and activation, were reduced, while *Cxcr2* and *Cxcr4*, involved in neutrophil trafficking, were upregulated (Figure S4A). It is important to note that in the single‐cell experiments, descriptive such as “higher” and “lower” refer to observed expression trends and do not reflect statistical significance; this interpretation applies throughout the manuscript.

### 2.2 FDE Exposure Reduces Inflammatory Eosinophil Numbers and Suppresses Lung IL‐5 and IL‐13 Levels

**FIGURE 3:**
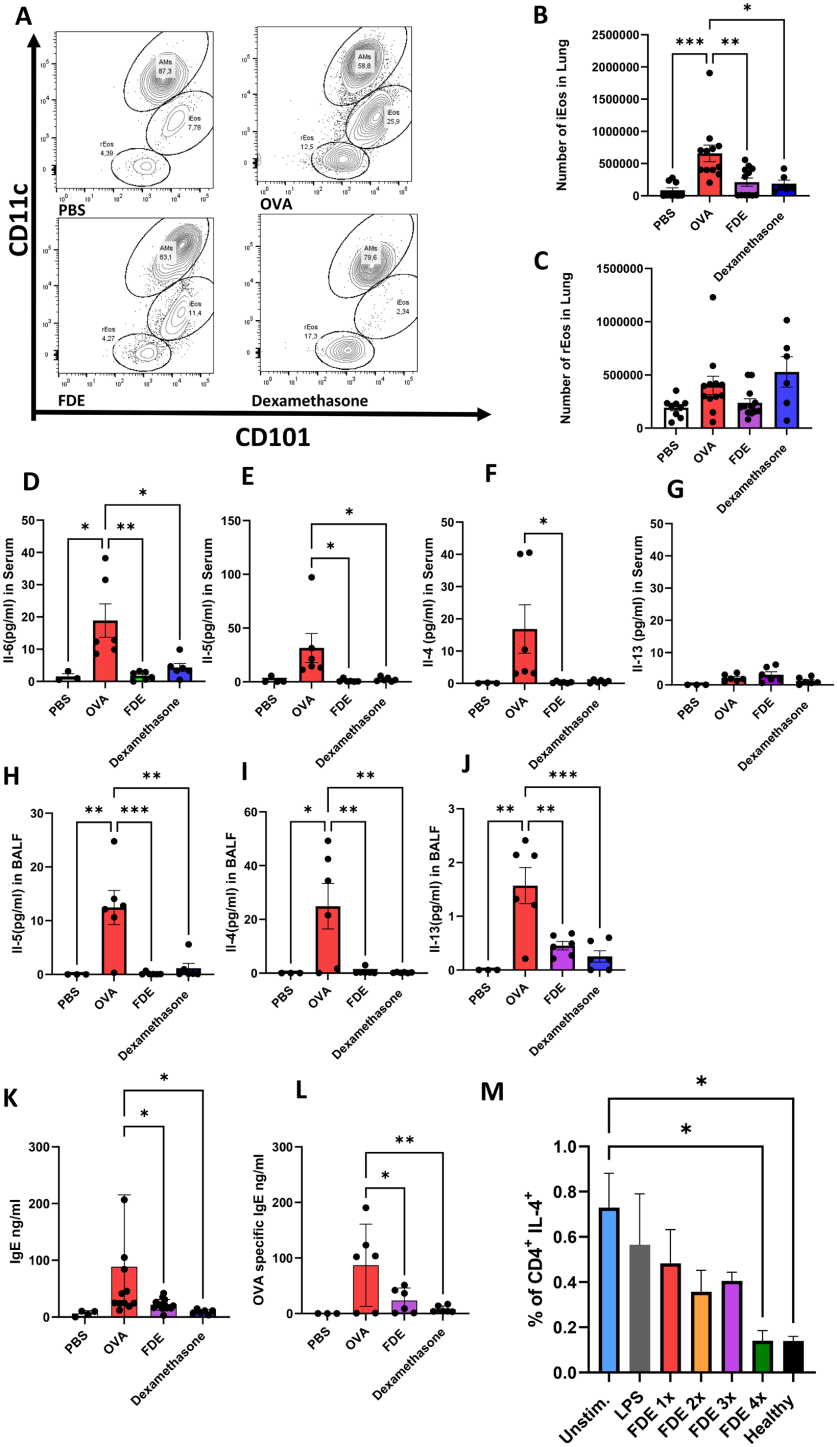
FDE diminishes inflammatory eosinophil counts and downregulates IL‐5 and IL‐13 levels in the lungs. (A) Contour plots of lung cells from PBS (upper left panel), OVA (upper right panel), FDE (lower left panel), and Dexamethasone (lower right panel) treated mice. SiglecF and CD125 were used to discriminate eosinophils from other lung cells. iEOS were identified by expression of CD101 and CD11c while rEOS stained negative for these markers. Alveolar macrophages expressed elevated levels of CD11c. (B) Quantification of pulmonary iEOS numbers, (C) quantification of pulmonary rEOS numbers upon PBS‐ (white bar), OVA‐challenged (red bar), and Farm dust exposed (FDE) (purple bar), or i.p. dexamethasone treatment (blue bar). Data shown are the mean ± SEM; *n* = 6–12. Data were analyzed by ANOVA followed by a Tukey test; *p < 0.05, **p < 0.01, ***p < 0.001. (D) Serum level of cytokine IL‐6 (pg/mL), (E) serum level of IL‐5 (pg/mL), (F) Serum level of IL‐4 (pg/mL), (G) serum level of IL‐13, (H) BALF level of IL‐5 (pg/mL), (I) BALF level of IL‐4 (pg/mL), (J) BALF number of IL‐13 (pg/mL), (K) Ig‐E concentration in serum (pg/ml), upon PBS‐ (white bar), OVA‐challenged (red bar), and farm dust exposure (FDE) (purple bar), or i.p. dexamethasone treatment (blue bar). Values shown are the mean concentration of Ig‐E ± SEM measured by ELISA. Data were analyzed by ANOVA followed by Tukey's test; *p < 0.05. Values are the mean ± SEM from *n* = 4–13 isolations. (L) OVA‐specific Ig‐E concentration in serum (pg/mL), upon PBS‐ (white bar), OVA‐challenged (red bar), and FDE (purple bar), or i.p. dexamethasone treatment (blue bar). Values shown are the mean concentration of Ig‐E ± SEM measured by ELISA. Data were analyzed by ANOVA followed by Tukey's test; *p < 0.05. Values are the mean ± SEM from *n* = 3–6 isolations. (M) Percentage of Th2 (IL‐4^+^CD4^+^) cells in asthmatic PBMCs under various stimulation conditions. Conditions include unstimulated cells (blue bar), cells stimulated with farm dust extract (FDE) once (red), twice (orange), three times (purple), or four times (green) at 24‐h intervals, and cells stimulated three times with LPS (gray). As a comparison, Th2 cell percentages in healthy donor PBMCs are shown (black bar). Data are presented as mean ± SEM; *n* = 2–4. Statistical analysis was performed using one‐way ANOVA followed by Tukey's multiple comparisons test; *p < 0.05.

Pulmonary eosinophils consist of two distinct populations—resident eosinophils (rEOS) and inflammatory eosinophils (iEOS)—with iEOS being recruited during pulmonary inflammation and playing a key role in allergic responses [16, 17]. We identified a population of SiglecF+CD125intCD101lo as rEOS in lung tissue and iEOS as SiglecF+CD125intCD101hi (Figure 3A). OVA exposure resulted in the accumulation of iEOS (Figure 3B). Additionally, we observed that FDE significantly reduced OVA‐induced inflammatory eosinophils (Figure 3B), but not rEOS (Figure 3C), suggesting that FDE attenuates allergic inflammation by targeting eosinophilic inflammation.

FDE significantly reduced levels of key cytokines involved in the pathogenesis of allergic asthma such as IL‐4, IL‐5, and IL‐13 in BALF and IL‐4 and IL‐5 in serum, which are critical for eosinophil recruitment and type 2 inflammation [18, 19]. FDE also reduced IL‐6 levels in serum, compared to OVA‐treated controls, and these effects were comparable to dexamethasone treatment (Figure 3D–J). Notably, IL‐10, IL‐9, and IL‐17F were below detection limits in both BALF and serum samples, indicating limited involvement in the observed inflammatory response. While IL‐17A and TNF‐α levels remained stable in serum, an increase in IL‐17A in BALF following FDE treatment was found (Figure S2).

IgE is a central mediator of allergic inflammation and plays a key role in eosinophil activation through Th2 cytokines [20, 21]. Supporting this notion, both total and OVA‐specific IgE levels were significantly reduced in FDE‐treated mice compared to OVA‐treated controls (Figure 3K,L).

To complement the findings in human samples, we evaluated Th2 cells via flow cytometry following in vitro treatment of PBMCs from adult asthmatic donors with FDE. The results showed a reduction in the percentage of IL‐4^+^ (Th2) cells with repeated FDE stimulation. This reduction was not seen after LPS stimulation, indicating that the effect is not LPS‐dependent. Notably, Th2 cell frequencies in the FDE 4× condition approached the levels observed in healthy donors. These findings suggest that FDE may specifically modulate Th2‐associated responses in human PBMCs and are consistent with the IL‐4 reductions observed in mouse serum and BAL fluid (Figure 3M).

### 2.3 FDE Enhances AREG Expression and Reduces MHC Class II in Epithelial Cells

**FIGURE 4:**
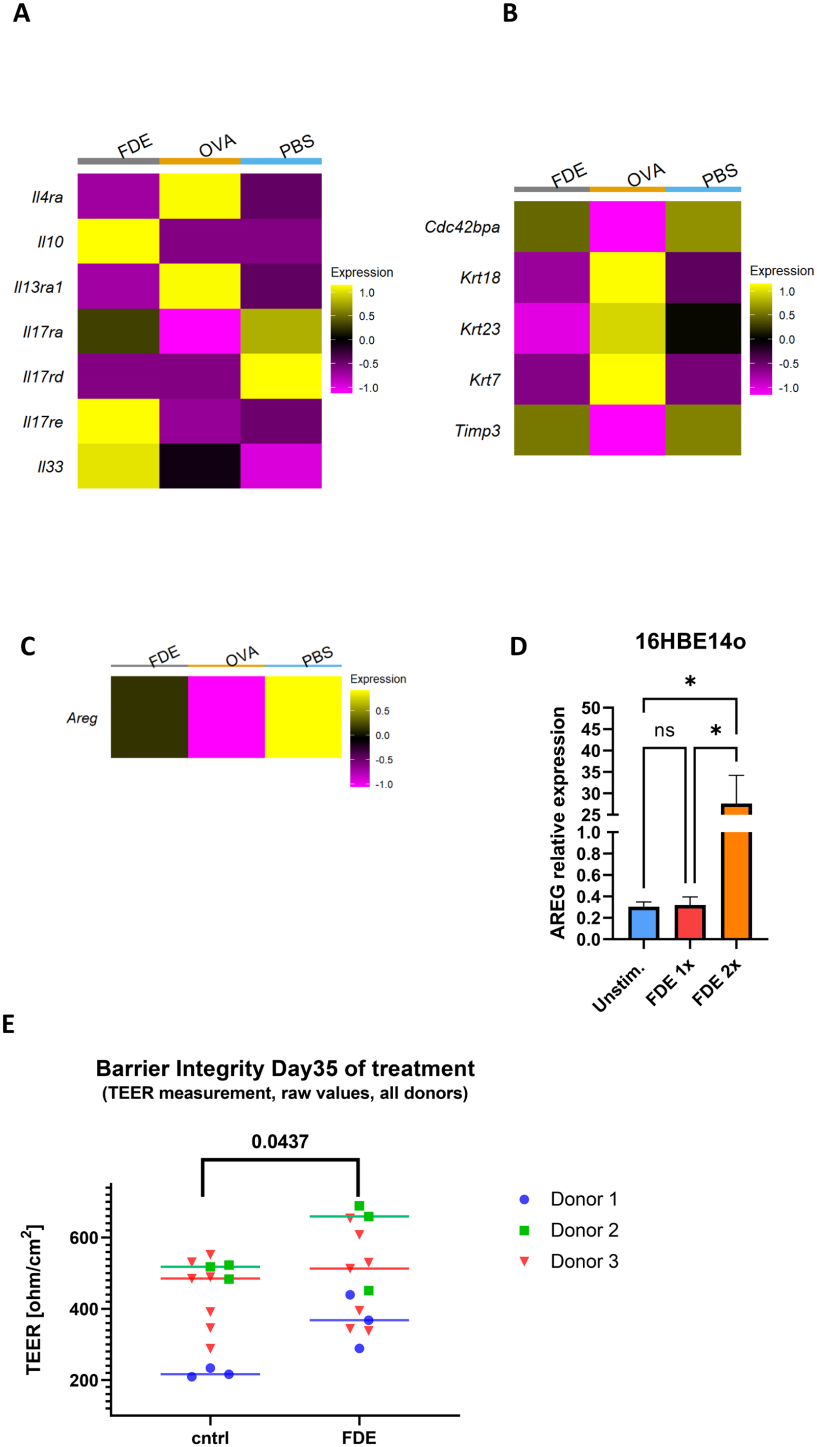
FDE enhances AREG expression while downregulating MHC Class II in epithelial cells. (A) Heat map analysis in the AT2 population based on the scaled gene expression profiles of key cytokines and their receptors. (B) Heat map analysis in the AT2 population based on the scaled gene expression profiles of key tissue remodeling and repair. (C) Heat map analysis of *Areg* in club cells. (D) 16HBE14o Cells were isolated upon starvation, treated once or twice at 24‐h intervals with FDE, and analyzed for RNA content. Data show an abundance of *Areg*. Values shown are the mean ± SEM from *n* = 3–6 isolations. Data show an abundance of mRNA reported to ß‐Actin. Statistical significance was assessed using a *t*‐test *p < 0.05. (E) Transepithelial electrical resistance (TEER) was measured in primary human bronchial epithelial cells (HBECs) derived from three independent donors to assess epithelial barrier integrity following treatment from day 0 to day 35 of differentiation at ALI with FDE. Each dot represents one technical replicate (color‐coded by donor), with horizontal lines indicating the associated mean value. Statistical significance and *p*‐value were derived using a paired one‐sided t‐test over the *n* = 3 mean donor values.

Single‐cell analysis of lung tissue revealed that FDE treatment notably altered the composition of epithelial cell populations, with a visible reduction in the proportion of AT2 cells compared to PBS (Figure S3A,B). To investigate the immune modulation induced by FDE, we examined key signaling pathways in alveolar type 2 (AT2) epithelial cells by single‐cell analysis, which are pivotal in mediating lung immune responses. FDE treatment normalized the expression of the IL‐4 and IL‐13 receptors, *Il‐13ra1* and *Il‐4rα*, in OVA‐challenged animals, reducing type 2 immune responses, while increasing the expression of the anti‐inflammatory cytokine IL‐10 (Figure 4A). These changes suggest that FDE mitigates allergic inflammation already at the epithelial level. The *Cdc42pba* gene, associated with tissue healing and epithelial stability, maintained expression levels similar to PBS in FDE‐exposed AT2 cells, while it was downregulated in OVA‐challenged animals (Figure 4B). This downregulation corresponded to a reduction in the expression of keratins (*Krt18*, *Krt23*, *Krt7*) after FDE treatment. Additionally, FDE exposure increased the expression of *Timp3*, a regulator of matrix metalloproteinases involved in tissue remodeling and repair [22] (Figure 4B).

Although Major Histocompatibility Complex (MHC) II molecules are traditionally associated with professional antigen‐presenting cells like dendritic cells and macrophages, airway epithelial cells can also express MHC‐II, particularly in inflammatory conditions like asthma [23, 24]. FDE treatment resulted in a decrease in the mRNA levels of MHC‐II genes (*H2ab1* and *H2eb1*) in AT2 epithelial cells, indicating potential reduced antigen presentation capabilities; however, this observation is based solely on transcriptional data and requires protein‐level and functional validation (Figure S4B). While *Muc5b* expression in goblet cells remained unchanged, *Muc5ac* expression in club cells was markedly reduced following FDE exposure (Figure S4C,D). This was further supported by PAS staining, which revealed a reduction in the PAS‐positive area in FDE‐treated mice compared to OVA‐treated controls, with this reduction being comparable to dexamethasone treatment (Figure S5A). These findings suggest that FDE not only modulates immune responses by downregulating *Mhc‐II* but also reduces mucus production in club cells and overall airway mucin content.

Building on this understanding, we investigated immunoregulatory pathways influenced by epithelial cells, including Amphiregulin (*AREG*), which is known to support immune tolerance and tissue repair. Among the epithelial cell types analyzed, *AREG* expression was detected solely in club cells and was upregulated following FDE treatment (Figure 4C). To model this response in vitro, we used 16HBE cells as a surrogate for club cells and similarly observed increased *AREG* expression after FDE stimulation. Using the 16HBE cell line, we conducted a starvation assay by depriving cells of serum (absence of FBS) to induce stress and disrupt the epithelial barrier, revealing a significant upregulation of *AREG* mRNA levels in FDE‐treated cells compared to untreated controls, suggesting a potential role for AREG in tissue repair under these conditions (Figure 4D). To assess the impact of FDE on epithelial barrier integrity, we measured transepithelial electrical resistance (TEER) in primary human bronchial epithelial cells derived from three independent donors. Treatment with FDE over 35 days of differentiation at ALI resulted in increased TEER values compared to control conditions (*p* = 0.04, paired one‐sided *t*‐test), indicating enhanced barrier function (Figure 4E).

We investigated epithelial mediators, including IL‐25, TSLP, and IL‐33, following FDE treatment and found that only IL‐33 showed a significant increase in expression (Figure 4A). As IL‐33 is known to promote AREG release and influence regulatory T cell (Treg) activation [25, 26] the observed upregulation of *IL‐33* suggests that FDE may impact immune pathways involved in epithelial stability and immune regulation.

### 2.4 FDE Exposure Modulates the Immune Response by Regulating CTLA‐4 Expression on Treg Cells

**FIGURE 5:**
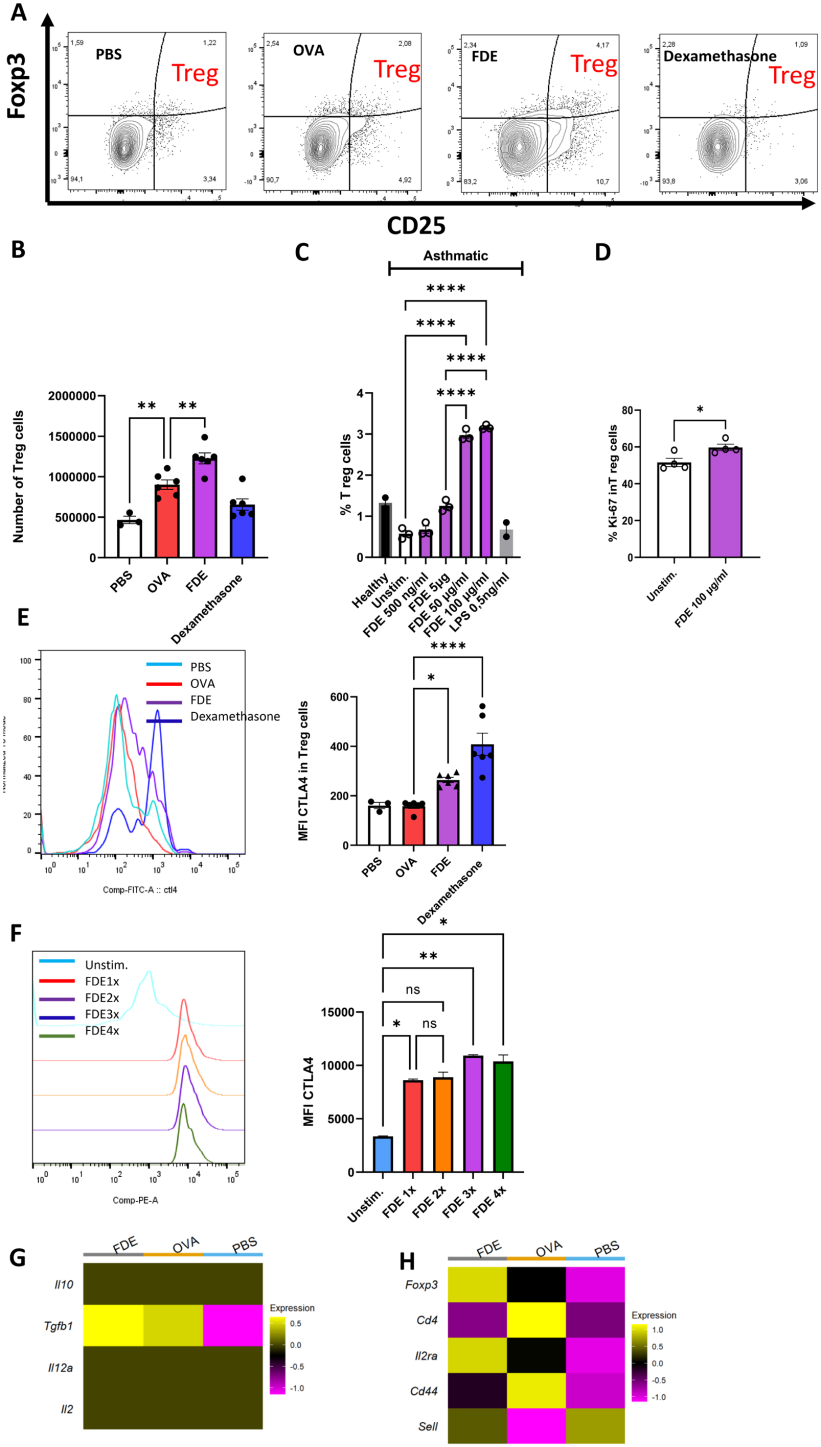
FDE attenuates the immune response by upregulating CTLA‐4 expression on regulatory T cells (Tregs). (A) Contour plots of lung cells from PBS, OVA, FDE, and Dexamethasone‐treated mice. CD3 and CD4 were used to discriminate T‐cells from other lung cells. Treg was identified by the expression of CD25 and Foxp3 double‐positive cells. (B) Quantification of pulmonary Treg numbers upon PBS‐ (white bar), OVA‐challenged (red bar), and FDE (purple bar), and Dexamethasone‐treated mice (blue bar). Data shown are the mean ± SEM; *n* = 3–6. Data were analyzed by ANOVA followed by a Tukey test; **p < 0.01. (C) Percentage of Treg cells in PBMCs isolated from asthmatic donor upon stimulation with PBS‐ (white bar), and different concentrations of Farm dust extract (FDE) (purple bars) 500 ng/mL, 50 μg/mL, 5 μg/mL, 100 μg/mL of farm dust extract or 0.5 ng/mL LPS for 24 h (gray bar). Treg cell percentages from unstimulated healthy donor PBMCs are shown for comparison (black bar). Data shown are the mean ± SEM; *n* = 2–3. Data were analyzed by ANOVA followed by a Tukey test; *p < 0.05, **p < 0.01. (D) Human PBMCs from healthy donors were stimulated once with 100 μg/mL FDE. Ki‐67 expression in Treg cells was measured by flow cytometry to assess proliferation. Data represent mean ± SEM from *n* = 4 donors. *p* < 0.05, one‐way ANOVA with Tukey's post hoc test. (E) Median fluorescence intensity (MFI) of CTLA‐4 on Treg cells upon PBS‐ (white bar), OVA‐challenged (red bar), and FDE (purple bar), and Dexamethasone‐treated mice (blue bar). Data shown are the mean ± SEM; *n* = 3–6. Data were analyzed by ANOVA followed by a Tukey test; *p < 0.05, ****p < 0.0001. (F) MFI of CTLA‐4 expression on Treg cells, asthmatic PBMCs (unstimulated) or 1 time stimulated (red), 2 times stimulated (orange), 3 times stimulated purple, and 4 times stimulated (green) with FDE at 24‐h intervals. Data shown are the mean ± SEM; *n* = 2. Data were analyzed by ANOVA followed by a Tukey test; *p < 0.05. (G) Heat map analysis in Treg cells based on scaled gene expression profiles of key immunological markers. (H) Heat map analysis in the T cell population based on the scaled gene expression profiles of key immunological markers.

Tregs act as key regulators of inflammation by suppressing the pathways that lead to immune cell activation, recruitment, and survival. They achieve this through cytokine modulation, checkpoint inhibition, and indirect effects on tissue repair [27, 28, 29]. In our study, the percentage and number of pulmonary Treg cells were significantly elevated in FDE‐treated animals compared to both PBS and OVA‐induced mice. Interestingly, this increase in Treg cells was not observed in the dexamethasone‐treated group (Figure 5A,B).

To complement our murine findings in human samples, isolated PBMCs derived from patients with asthma were treated in vitro with varying doses of FDE following T cell activation. The result demonstrated that FDE treatment significantly increased the proportion of Treg cells in a dose‐dependent manner (Figure 5C). In contrast to unstimulated PBMCs from asthmatic donors, which showed low frequencies of Tregs, healthy donor PBMCs exhibited higher baseline levels of Tregs, indicating a more balanced immune profile. To rule out the contribution of endotoxin (LPS) to the observed immunomodulatory effects of FDE, we treated PBMCs with 0.5 ng/mL of purified LPS—the concentration present in 100 μg/mL of FDE. This treatment did not alter Treg cell numbers, indicating that the FDE‐induced expansion of Tregs occurs independently of its LPS content (Figure 5C). To investigate the mechanism behind the Treg increase, we measured Ki67 expression in Tregs from healthy donors. FDE treatment elevated the proportion of Ki67^+^ Tregs, indicating that enhanced proliferation contributes to their expansion (Figure 5D).

Treg cells constitutively express CTLA‐4, which blocks the priming and activation of naïve CD4+ T (Tconv) cells to antigen‐presenting cells (APC)s [30]. We assessed CTLA‐4 expression in pulmonary Treg cells using flow cytometry. Interestingly, we observed a significant upregulation of CTLA‐4 in FDE‐treated mice compared to both the PBS control and OVA‐induced mice (Figure 5E). Notably, dexamethasone treatment led to an even higher increase in CTLA‐4 expression, despite no significant change in the number of Treg cells. Similarly, in human asthmatic PBMCs, repeated FDE stimulation resulted in increased CTLA‐4 expression in Treg cells (Figure 5F).

Treg‐mediated suppression involves cell contact‐dependent and humoral mechanisms, utilizing CTLA‐4, IL‐10, TGF‐β, IL‐12α, and IL‐22 [26]. We therefore assessed the humoral properties of murine Treg cells using single‐cell analysis. Data revealed that only TGF‐β significantly increased in Tregs in FDE‐treated animals in comparison to the OVA‐induced group (Figure 5G).

A heat map of gene expression profiles from single‐cell analysis revealed significant upregulation of *Foxp3* and *Il‐2rα* (CD25) in FDE‐treated mice (Figure 5H). These markers are critical for Treg cell development, survival, and function [26, 31]. This regulatory enhancement is complemented by the observed upregulation of *Sell* (CD62L gene) (Figure 5H), a marker linked to naive and central memory T cells, and a key contributor to Treg homing [32, 33]. Importantly, CD62L + Tregs have been shown to exhibit superior suppressive capabilities, which may be attributed, in part, to their ability to sustain higher levels of CTLA‐4 [28]. Conversely, the downregulation of *Cd44* (CD44 gene), a marker typically associated with activation and effector function, further supports a shift toward a more regulated immune phenotype (Figure 5G). Moreover, single‐cell analysis revealed enhanced cell–cell communication involving antigen‐presenting cells—macrophages and dendritic cells (DCs)—and regulatory T cells (Tregs) following FDE exposure (Figure S3E,F), suggesting increased potential interactions among these immune populations.

### 2.5 FDE Treatment Downregulates DC Function

To directly evaluate whether FDE acts as an APC inhibitor, we identified and analyzed the DC population using flow cytometry (Figure S3C,D).

**FIGURE 6:**
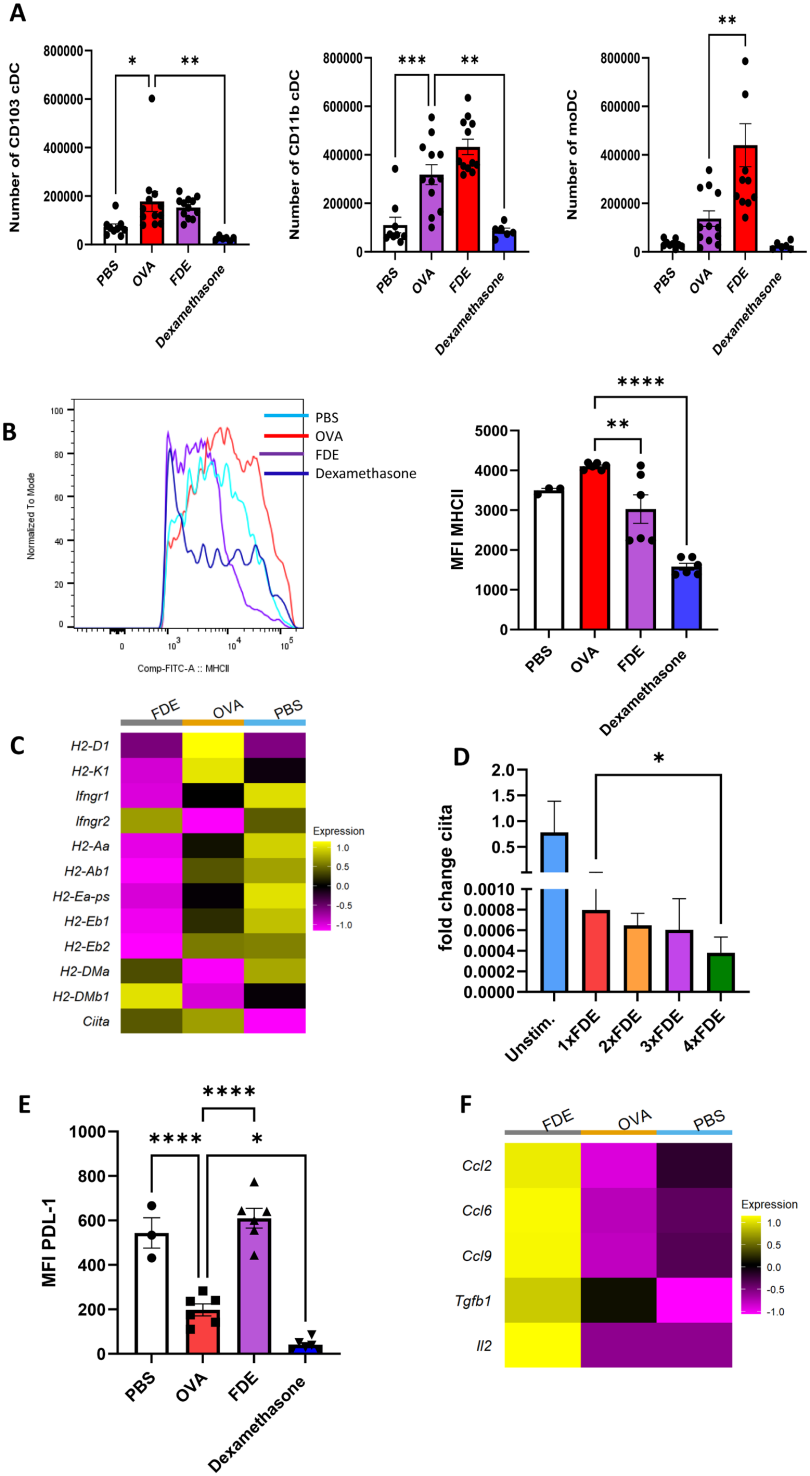
FDE impairs dendritic cell function. (A) Quantification of pulmonary left to right CD103 + cDC, CD11b + cDC, and moDC numbers upon PBS‐ (white bar), OVA‐challenged (red bar), and FDE (purple bar), Dexamethasone‐treated mice (blue bar). Data shown are the mean ± SEM; *n* = 3–6. Data were analyzed by ANOVA followed by a Tukey test; *p < 0.05 ***p < 0.001. (B) MFI of MHC‐II at the surface of DC cells upon PBS‐ (white bar), OVA‐challenged (red bar), and FDE (purple bar), Dexamethasone‐treated mice (blue bar). Data show ΔMFI ± SEM; *n* = 3–6. Data were analyzed by ANOVA followed by a Tukey test; **p < 0.01, ****p < 0.0001. (C) Heat map analysis in DC cells based on gene expression profiles of key MHC‐I and MHC‐II genes. (D) Cells were isolated from asthmatic donors, unstimulated or 1 time stimulated (red), 2 times stimulated (orange), 3 times stimulated purple, and 4 times stimulated (green) with FDE at 24‐h intervals and analyzed for RNA content. Data show an abundance of *CIITA*. Values shown are the mean ± SEM from *n* = 4 isolations. Data show an abundance of mRNA reported to *ß‐Actin*. Statistical significance was assessed using a t‐test *p < 0.05. (E) Expression of PDL‐1 at the surface of DC cells upon PBS‐ (white bar), OVA‐challenged (red bar), and FDE (purple bar). Data show ΔMFI ± SEM; *n* = 3–6. Data were analyzed by ANOVA followed by a Tukey test; ***p < 0.001, ****p < 0.0001. (F) FDE may modulate Treg cell recruitment by enhancing the secretion of macrophage‐derived cytokines and chemokines. Heat map analysis in the macrophage population based on the scaled gene expression profiles of key immunological markers.

To explore the mechanisms behind the increased accumulation of CTLA‐4‐expressing Treg cells in the lungs of FDE‐treated animals compared to OVA‐induced mice, we assessed the homing of different dendritic cell (DC) subsets to the lung. DCs were identified based on surface marker expression (Figure S1C). OVA challenge significantly increased the number of CD11b + conventional dendritic cells (cDCs). In contrast, exposure to FDE caused a slight increase in both CD11b + cDCs and monocyte‐derived dendritic cells (moDCs), while the numbers of CD103 + cDCs remained unchanged (Figure 6A). Interestingly, while FDE did not affect the total number of CD103 + cDCs, it did increase CD103 expression within the DC population (Figure S1D). Although FDE slightly elevated CD11b + cDCs and moDCs, DCs from FDE‐treated mice exhibited lower MHC‐II expression compared to the OVA‐induced group (Figure 6B). By downregulating MHC‐II expression, FDE might limit the ability of DCs to activate naïve CD4 + T cells, thereby reducing overall T cell activation and inflammatory responses. This creates a less stimulatory environment, preventing the excessive activation of pro‐inflammatory Th2 cells commonly seen in allergic asthma. Similarly, dexamethasone treatment reduced MHC‐II expression on DC populations without increasing the overall number of DCs (Figure 6B).

Moreover, we generated a heat map based on the gene expression profiles of key antigen presentation markers on DCs. The data confirmed that FDE‐treated mice downregulated the MHC‐I associated genes, including *H2‐k1* and *H2‐d1*, in comparison to OVA‐induced mice. Class II transactivator (*CIITA*), a key transcriptional regulator of MHC‐II expression, appeared modestly reduced in FDE‐treated mice based on the single‐cell heatmap; however, this reflects a trend rather than a definitive downregulation. Similarly, some of the MHC‐II genes, not all, (H*2‐aa*, *H2‐ab1*, *H2‐ea‐ps*, *H2‐eb1*, and *H2‐eb2*), were downregulated after FDE treatment. In turn, H2‐DMa and H2‐DMb1 were upregulated in FDE‐treated animals (Figure 6C). This pattern of gene expression suggests that FDE treatment may lead to selective modulation of antigen presentation capabilities in dendritic cells. However, the upregulation of *H2‐dma* and *H2‐dmb1*, which participate in MHC‐II antigen processing, suggests that FDE treatment might still allow for some level of antigen presentation, in a manner that supports immune tolerance rather than activation. This selective modulation might be a mechanism by which FDE treatment reduces the severity of allergic responses in the lung. We assessed the gene expression profile on other cell types, including macrophages and T cells, and observed that this modulation was specific for DCs (Figure S4E,F).

To complement our murine findings in humans, isolated PBMCs from asthma patients were treated in vitro with an increasing treatment cycle of FDE, followed by measurement of *CIITA* mRNA expression. FDE treatment resulted in greater downregulation of *CIITA* than unstimulated (Figure 6D).

To investigate whether the immune‐regulatory effects observed in antigen‐presenting cells extended to pathways, we measured the PD‐L1 expression on murine DCs by flow cytometry. PD‐L1 is known to interact with PD‐1 on T cells, leading to the inhibition of T cell activation and the promotion of immune tolerance [34, 35]. PD‐L1 expression on DCs decreased in OVA‐induced mice, which is typically associated with a reduced inhibitory signal that could lead to heightened T‐cell activation and allergic inflammation (Figure 6E). However, after FDE treatment, this decrease in PD‐L1 was reversed, bringing the levels back to those observed in PBS‐treated control mice (Figure 6E).

### 2.6 FDE Treatment Promotes the Secretion of Macrophage‐Derived Cytokines and Chemokines

First, we assessed the impact of farm dust extract on alveolar macrophages (AM) and interstitial macrophages (IM). Interestingly, FDE treatment did not result in any significant changes in the number of these macrophage populations (Figure S4G,H).

The recruitment of Treg cells to sites of inflammation is driven by cytokines, including IL2 and TGF‐β, and chemokines, including CCL9 (Macrophage Inflammatory Protein‐1γ), CCL6 (Macrophage Inflammatory Protein‐1α or MIP‐1α), and CCL2 (Monocyte Chemoattractant Protein‐1 or MCP‐1), which act as chemoattractants [36].

To determine if macrophages release chemoattractants following FDE treatment, we used single‐cell analysis. Data revealed that genes associated with the recruitment of Treg cells, including *Il2*, *Tgf‐β*, *Ccl9*, *Ccl6*, and *Ccl2*, were upregulated in macrophages of FDE‐treated mice in comparison to OVA‐challenged mice (Figure 6F). This observation aligns with the higher number of Treg cells observed in the lungs of FDE‐treated mice.

Transcriptomic profiling of TGF‐β1–associated genes revealed differential expression patterns in FDE‐treated samples, suggesting that FDE may influence TGF‐β1–related immune signaling pathways. These gene expression changes suggest that FDE modulates components of the TGFB1 signaling pathway; however, the functional implications of this modulation remain to be determined. While some gene sets have been associated with asthma symptom severity in prior studies, our findings are exploratory and do not support causal conclusions [29] (Figure S7).

## 3 Discussion

This study demonstrates that FDE effectively reduces hallmark asthma features, including airway eosinophilia, small and total airway hyperresponsiveness (AHR), mucus secretion, airway inflammation, and IgE production in OVA‐sensitized mice. Notably, FDE treatment enhanced epithelial barrier integrity, as evidenced by increased TEER and upregulation of AREG expression, indicating a potential barrier‐stabilizing effect. In line, FDE modulated the airway epithelium by promoting IL‐33 and IL‐10 release, which may support Treg‐mediated immune regulation, likely driven by the diverse microbial components of the extract. We did not perform a control experiment to exclude the contribution of LPS. However, literature shows that LPS alone primarily induces pro‐inflammatory cytokines like IL‐6 and IL‐8 in AT2‐like cells, while IL‐33 induction typically requires additional stimuli such as allergens or mechanical stress [23, 37, 38]. This supports the notion that the effects observed with farm dust extract may be distinct from those of LPS alone.

IL‐33 and AREG are mediators with context‐dependent roles—while IL‐33 is widely recognized for promoting type 2 inflammation and is a therapeutic target in asthma, it can also support tissue repair and enhance Treg function under certain conditions. Similarly, AREG has been implicated in both inflammatory and regulatory processes [39, 40]. In our study, their expression appears to be associated with epithelial barrier stabilization and regulatory immune responses, rather than promoting tissue remodeling [41, 42]. Importantly, IL‐33 biology differs between mice and humans. In mice, IL‐33 is mainly expressed by AT2 cells, whereas in humans, it is found in a broader range of cells, including epithelial and endothelial cells [43, 44].

On the other hand, FDE treatment was accompanied by an increase in neutrophils and IL‐17 in the BALF, raising important considerations regarding the underlying mechanisms and potential implications. However, the phenotypic characteristics of the neutrophils observed in this study—such as reduced CD11b (Itgam) expression—align with findings from Stein et al. regarding neutrophil phenotypes in the Amish population with a significantly lower prevalence of asthma [12] (Figure S4A). Additionally, the expression of CD101 may indicate a shift toward a less activated phenotype [45, 46, 47, 48] with dampened inflammatory capacity of neutrophils in the airways following FDE exposure. Furthermore, the increased expression of CXCR2 and CXCR4 on neutrophils suggests enhanced responsiveness to chemokines that mediate neutrophil trafficking, which could contribute to the accumulation of neutrophils in the airways. Alternatively, these neutrophils might reflect low‐grade inflammation after frequent applications of FDE.

FDE treatment significantly reduced small and total airway resistance but not large airway resistance, highlighting its targeted effects on the distal airways. Small airways are more prone to obstruction due to their smaller caliber and greater sensitivity to inflammation [49, 50].

FDE stabilizes the epithelial barrier by decreasing antigen presentation and selectively reducing *Muc5ac* expression, a marker of mucus overproduction while preserving *Muc5b* expression, which is essential for effective mucus clearance. While corticosteroids like dexamethasone remain a cornerstone of asthma treatment due to their anti‐inflammatory properties, their effects on specific pathways are limited. Notably, dexamethasone does not fully prevent IL‐17A‐induced epithelial barrier disruption, goblet cell metaplasia, or mucus overproduction, highlighting gaps in its therapeutic scope [51, 52].

In our mouse single‐cell RNA‐seq dataset, AREG expression was specifically increased in club cells. Based on this observation, we employed the human bronchial epithelial cell line 16HBE as an in vitro model to assess whether FDE modulates epithelial repair‐associated responses in the bronchial compartment. FDE treatment resulted in elevated AREG mRNA expression in 16HBE cells, accompanied by a corresponding increase in TEER, indicating improved epithelial barrier function. While these results indicate a potential pro‐repair effect of FDE, we acknowledge that direct evidence linking AREG to barrier restoration is lacking. Future studies using targeted AREG knockdown will be crucial in establishing its mechanistic role in mediating the observed effects.

Previous studies have demonstrated that AREG enhances Treg suppressive function, which is crucial for controlling local inflammation [25, 31]. These findings prompted further investigation into potential mediators, which revealed an associated increase in IL‐33 levels. IL‐33, an epithelial alarmin, promotes AREG release and enhances Treg activation [25, 26, 31, 40, 53]. Upon activation, CD4+ Treg cells then migrate to the site of inflammation, where exposure to AREG enhances their suppressive abilities [54]. Interestingly, among other epithelial alarmins evaluated, including IL‐25 and TSLP, only IL‐33 showed increased expression following FDE treatment. The interplay among IL‐33, AREG, and Tregs suggests that FDE mitigates allergic inflammation not only by stabilizing epithelial function but also by enhancing Treg‐mediated immune regulation.

In the context of asthma and allergic diseases, epithelial MHC‐II expression can activate T‐helper cells, promoting the release of cytokines such as IL‐4, IL‐5, and IL‐13, which drive eosinophilic inflammation and mucus overproduction—hallmark features of allergic asthma [55]. Our findings suggest that FDE exposure has a comparable effect to dexamethasone in downregulating MHC‐II expression in a murine model of asthma. While dexamethasone is known to upregulate FOXP3 expression and enhance regulatory T cell (Treg) numbers in some asthma patients [56], our results did not show a significant increase in Treg cells in dexamethasone‐treated mice compared to OVA‐challenged controls. Interestingly, several immunoregulatory genes, including *Tgfb1*, *Foxp3*, and *Il2ra* (*CD25*), were already upregulated in OVA‐induced mice compared to PBS controls and were further enhanced upon FDE treatment. This may suggest that FDE may amplify regulatory pathways that are endogenously activated during allergic inflammation, potentially contributing to its anti‐inflammatory effect by boosting Treg‐associated responses in an already inflamed environment. In addition, both FDE‐exposed and dexamethasone‐treated mice showed increased expression of CTLA‐4 in Treg cells. This indicates that FDE not only promotes Treg expansion but also boosts their capacity to suppress effector T cell activation [30, 57]. This aligns with the observed decrease in antigen presentation by MHC‐II in dendritic cells, supporting a shift toward a more tolerogenic immune environment.

The downregulation of *CIITA* in human PBMCs supports this mechanism. In addition to reducing antigen presentation, FDE treatment restored PD‐L1 expression on DCs to levels comparable to PBS controls, counteracting the reduction observed with OVA treatment. In contrast, dexamethasone has been shown to suppress inflammatory responses but does not restore PD‐L1 expression to baseline levels, highlighting a key difference in the immunomodulatory effects of FDE [52, 58]. The elevation of PD‐L1 expression in DCs suggests that FDE not only reduces their capacity to present antigens but also enhances their ability to engage in immunosuppressive mechanisms.

Notably, we did not detect the presence of dexamethasone or other corticosteroids in the FDE extract, ruling out the possibility that FDE's effects are due to contamination with exogenous steroids (Table S1). Unlike dexamethasone, which is known to skew the immune response by downregulating Th1 cytokines, in some cases even promoting Th2 [4], FDE treatment led to a dampening of Th2 responses without a corresponding shift in Th1 activity [12, 59], as evidenced by the lack of changes in tumor necrosis factor‐alpha (TNF‐α) levels and undetectable levels in interferon‐gamma (IFN‐γ) in BALF. This selective suppression of Th2 cells, without altering Th1 pathways, highlights a distinct advantage of FDE. By providing targeted anti‐inflammatory effects while avoiding the broad immunosuppression commonly associated with corticosteroid treatments, FDE demonstrates its potential as a more specific therapeutic option for managing allergic inflammation. This approach could offer an alternative for patients who do not respond to corticosteroids or experience significant side effects. However, repeated inhalation of unprocessed environmental dusts carries potential risks, including airway irritation, inflammation, or unintended immune activation, particularly in vulnerable populations. Therefore, further investigation is needed to identify the active compounds in FDE and assess its potential side effects to ensure its safety and efficacy as a therapeutic option.

BV‐OM85 is composed of inactivated bacterial components and is known to enhance immune regulation and dampen airway inflammation through the expansion of Tregs along with CTLA4 and suppression of DC responses in the airways following allergen challenge [60, 61]. BV‐OM85 mediates anti‐inflammatory effects via IL‐10, while FDE does not alter IL‐10 levels in BALF or serum. This may stem from their composition differences—BV‐OM85, derived from Gram‐positive and Gram‐negative bacteria, contains LPS, which induces IL‐10 via TLR4, whereas FDE is an autoclaved extract from a microbe‐rich environment like cow shed dust [11, 62, 63]. In addition, BV‐OM85 promotes immune tolerance in asthma by increasing the number of tolerogenic CD103^+^cDCs [61]. In contrast, our findings indicate that FDE does not increase the number of CD103^+^ DCs in the airways. Despite this, FDE‐treated mice exhibited higher surface expression of CD103 on existing DCs, suggesting that while the quantity of these cells remains unchanged, their functional capacity may be enhanced.

Our study used a secondary preventive model, a common approach in preclinical asthma research. Several asthma therapies, including inhaled corticosteroids and biologics targeting IL‐5, IL‐4R, or IL‐33, have been tested in similar models to assess their ability to modulate airway inflammation [64, 65, 66]. While this does not reflect treatment of established disease, it provides early insight into therapeutic potential. A limitation of our study is the small number of allergic asthma donors of PBMC samples, which, despite showing consistent trends, may not capture the full spectrum of asthma phenotypes. Future studies should include larger cohorts to validate and extend these findings.

Collectively, these findings indicate that FDE modulates both structural and immune components of asthma, promoting epithelial repair and immune tolerance while suppressing inflammatory pathways. The validation of these effects in human PBMCs from asthmatic donors highlights the translational relevance of the data. Future studies should focus on identifying the active principles within FDE to develop novel non‐steroidal therapies targeting the multifaceted pathogenesis of asthma.

## 4 Methods

### 4.1 Mice

WT female Balb/c mice were purchased from Charles River Germany. All mice were maintained at the Helmholtz Zentrum München specific pathogen‐free facility and were regularly fed with sufficient water and food. Mice used for experiments were 8–12 weeks of age. Animal care was carried out in accordance with the regulations of the German animal welfare law. This study was reviewed and approved by the Government of Upper Bavaria (ROB‐55.2‐2532.Vet_02‐23‐27 and ROB‐55.2‐2532.Vet_02‐20‐96).

### 4.2 Experimental Allergic Asthma Models

The OVA‐induced asthma model was performed as described previously [67] with minor modifications (Figure 1A). Briefly, mice were immunized by intraperitoneal (i.p.) injection with OVA‐Alum (50 μg/1 mg) on days 0, 7, and 14, challenged intranasally (i.n.) with 100 μg OVA (in 25 μL PBS) on days 26, 27, and 28. The farm dust extracts (FDE) were prepared and analyzed as described [14]. The farm dust was collected by sweeping settled dust from ledges, windowsills, and elevated surfaces (≥ 1 m above ground level) within cow sheds, similar to previously published approaches [12]. For extraction, 2.5 g of farm dust (particle size 40–100 μm) were weighed into a 50‐mL centrifuge tube, sterile water (25 mL) was added, and shaken for 2 h at 1000 rpm at room temperature (RT). Next, the sample was centrifuged at 2500× *g* for 5 min at RT. First, the supernatant was filtered through two bottle‐top filtration systems (pore size 13 and 2 μm; cellulose membrane) to remove insoluble material. Then the prefiltered extract was filtered a third time through a bottle‐top filtration system (pore size 0.22 μm polyethersulfone (PES) membrane). After the filtration steps, FDEs were autoclaved with pressurized saturated steam for 15 min at 121°C. Then sterile FDEs were centrifuged with 20‐mL centrifugal concentrators (PES membrane, molecular weight cut‐off [MWCO]: 10 kDa; Sartorius, Göttingen, Germany) at 3500× *g* for 45 min at RT. Ten milliliters of sterile water were added to the obtained residue, containing molecules with a MWCO of > 10 kDa. The water was removed by centrifugation at 3500× *g* for 30 min at RT (removal of restrained small molecules; purification step). The purification step was repeated under the same conditions before the residue was resuspended in sterile water (3–6 mL). The resuspended FDEs (MWCO > 10 kDa) were filtered a fourth time through a bottle‐top filtration system (0.22 μm PES membrane). Finally, under sterile conditions, 3.0 mL of the sterile and concentrated DE was filled into a 5‐mL glass vial and freeze‐dried (Christ Epsilon 2‐6D LSC; Osterode am Harz, Germany). The lyophilized FDE was stored at −20°C. The LPS concentration in FDE was quantified as approximately 5 ng per mg of extract using the Pierce Chromogenic Endotoxin Quant Kit (based on the Limulus Amebocyte Lysate [LAL] method). FDE or PBS was administered to the animals intranasally at 32 mg/mL (in 25 μL PBS) on days 16, 19, 21, 23, 26, 27, and 28. The mice were sacrificed, and organs were harvested 24 h after the last administration for single‐cell analysis. In some experiments, in addition to the protocol in Figure 1A, dexamethasone (0.5 mg/kg) was administered intraperitoneally (Figure 1B).

### 4.3 Determination of AHR

Mice were anesthetized by i.p. injection of 50 μL Ketamin/Rompun (76 and 4.8 mg/mL respectively, Pfizer/Bayer). AHR was measured in anesthetized mice that were mechanically ventilated using a FlexiVent (SciReq) system as described [68]. Aerosolized Acetyl‐β‐methyl‐choline (methacholine) (0, 3, 6, 12, and 24 mg/mL; Sigma‐Aldrich) was generated by an ultrasonic nebulizer and delivered in‐line through the inhalation port for 10 s. Airway resistance was measured 2 min later.

### 4.4 Pulmonary Cell Isolation

Liberase TL (Roche) 0.25 mg/mL and DNase I 0.5 mg/mL (Sigma‐Aldrich) digests of the lungs were prepared to obtain single lung cell suspensions. The single cell suspension was prepared by mechanical disruption of the lungs using a 5‐mL syringe stamp and an additional 10 mL complete medium (complete medium: RPMI 1640 supplemented with 10% fetal bovine serum (FBS, PAA Laboratories, Pasching, Austria) heat‐inactivated, 100 units/mL Penicillin, 100 μg/mL Streptomycin, 2 mM l‐Glutamine) in the presence of 0.5 mg/mL DNase. The smoothing step was repeated twice with a subsequent step of washing with 5 mL of complete medium. The cell suspension was centrifuged for 10 min at 350× *g* at 4°C. The supernatant was discarded, and the pellet was re‐suspended in 3 mL red blood cell lysis buffer (Sigma‐Aldrich) for 3 min at room temperature (RT). The lysis was stopped by adding 30 mL of PBS, and the cell suspension was centrifuged for 5 min at 400× *g* at 4°C. After cell counting with trypan blue in a Neubauer counting chamber, the single cell suspension was used for further analysis.

### 4.5 Cell Phenotyping by Flow Cytometry

To block nonspecific staining by fluorochrome‐conjugated antibodies, cells were preincubated with Fc Block (BD Bioscience: 10 μg/mL) for 10–15 min on ice. For staining, cells were incubated for 30 min on ice in the dark with antibodies. The following anti‐mouse surface antigen antibodies were used for flow cytometry: lung eosinophils were identified using recently published gating strategies [17], anti‐CD125‐BV711, anti‐CD101‐PE, anti‐CD11c‐APC, and anti‐Siglec F‐BV421. Lung dendritic cells were identified using published gating strategies [69], anti‐MHCII‐FITC, anti‐CD103‐Percp‐Cy5.5, anti‐CD11c‐APC, and anti‐Siglec‐F‐BV421, eFluor (eF) 450‐labeled Abs against CD19, CD3e, and CD49b (DX5); anti‐CD11b‐BV480, anti‐PDL‐1‐BV605, and anti‐CD64‐BV711 (from Biolegend or BD Bioscience). To remove dead cells, Phycoerythrin (PE) CF594 labeled Fixable Viability Dye was used. T cells were identified by anti‐CD44‐APC, anti‐CD4‐PE‐Cy7, anti‐CD3‐BV421, anti‐CTLA4‐FITC, anti‐CD25‐BV711, and anti‐CD62L‐PE CF594 (from Biolegend or BD Bioscience). For intracellular staining, cells were fixed and permeabilized using the FOXP3/Transcription Factor Staining Buffer Set (eBioscience) and stained with anti‐FOXP3‐PE. Gating strategies for the identification of dendritic cells (DCs) are shown in Figure S1C. Macrophages and eosinophils were first excluded based on SiglecF expression. The remaining SiglecF^−^ population was further gated to exclude lineage‐positive cells (CD19^+^ B cells, CD3e^+^ T cells, CD49b^+^ NK cells, and Ly6G^+^ neutrophils), resulting in a lineage^−^ fraction used for dendritic cell analysis.

Dendritic cells (DCs) were identified within the lineage^−^ SiglecF^−^ population as CD11c^+^ MHC‐II^hi^ cells. DC subsets were further discriminated using CD103 and CD11b expression. CD103^+^ CD11b^−^ cells were classified as conventional dendritic cells type 1 (cDC1), while CD103^−^ CD11b^+^ cells included two populations: CD11b^+^ CD64^−^ cells corresponding to conventional dendritic cells type 2 (cDC2), and CD11b^+^ CD64^+^ cells corresponding to monocyte‐derived dendritic cells (moDCs). Phenotypic characterization of cells was performed on a FACSymphony A3 and LSRFortessa II flow cytometer (BD Biosciences). Depending on the target cell population, 30,000–400,000 events were acquired per sample and analyzed with FlowJo software (Version 10.10.0, Becton Dickinson, 2019).

### 4.6 Collection of Bronchoalveolar Lavage (BAL) Fluid and Differential Cell Counts

BAL fluid samples were obtained by cannulating the trachea, injecting 3 × 250 μL of ice‐cold PBS, and subsequently aspirating the BAL fluid. After red blood cell lysis, BAL fluid cells were washed once in PBS and counted using a Neubauer chamber (Assistant, Germany). Cell numbers were calculated using cell‐specific frequency of total and total cell counts/mL. To block nonspecific staining by fluorochrome‐conjugated antibodies, cells were preincubated with Fc Block (BD Bioscience: 10 μg/mL) for 10–15 min on ice. The following anti‐mouse surface antigen antibodies were used for flow cytometry: anti‐Siglec‐F‐APC‐Cy7, anti‐CD11c‐APC, anti‐Ly6G‐BV421, and anti‐CD4‐PE‐Cy7. Frequencies of BAL fluid cells were determined by FACSymphony. Gating strategies for the identification of alveolar macrophages, eosinophils, T cells, and neutrophils are shown in Figure S1F.

### 4.7 Lung Histology

Lung histological staining, detection, and quantification of mucus cell content were performed as described [70]. Slides were stained with periodic acid‐Schiff (PAS). PAS‐positive and PAS‐negative airways were counted by light microscopy, and the percentage of PAS‐positive airways was calculated to quantify mucus production [71, 72]. For the assessment of inflammatory cell infiltration, sections were stained with hematoxylin and eosin (H&E). Paraffin‐embedded lung tissue slides were first heated to melt the paraffin, rehydrated through graded alcohols, stained with H&E, dehydrated, and cover‐slipped. Histologic evaluation was performed at an original magnification of ×20.

### 4.8 Multiplex Assay

LEGENDplex pre‐defined mouse T helper cytokine panel assay from BioLegend was utilized according to the manufacturer's recommendations for analyzing bronchoalveolar lavage fluid (BALF) and serum samples. This panel allows simultaneous quantification of 12 mouse cytokines, including IFN‐γ, IL‐5, TNF‐α, IL‐2, IL‐6, IL‐4, IL‐10, IL‐9, IL‐17A, IL‐17F, IL‐22, and IL‐13. Cytokines were quantified by FACSymphony.

### 4.9 Peripheral Blood Mononuclear Cells (PBMCs) Isolation

Samples from asthma patients were provided by Ludwig‐Maximilians Universität and Asklepios Klinik Gauting according to proposal number BA173/2023, Ethics Committee vote number 19–629, or were purchased from DONAS GmbH. Exclusion criteria were a history of daily smoking for at least 1 year, use of oral steroids within the last 8 weeks, current use of specific inhalers, and being older than 65 years of age (Table S2). All donors gave informed consent. For DONAS GmbH, the donor authorization, production, storage, and transport are carried out in accordance with the “Guidelines for the collection of blood and blood components and the use of blood products” (drawn up by the German Medical Association in agreement with the Paul Ehrlich Institute in accordance with Sections 12a and 18 of the Transfusion Act) in the currently valid version. Peripheral blood mononuclear cell (PBMC) isolation was carried out following minor modifications based on the recommendations provided by STEMCELL Technologies. Lymphoprep (STEMCELL Technologies) was used in conjunction with gradient centrifugation for the isolation process.

### 4.10 PBMC Cell Stimulation and Cell Phenotyping by Flow Cytometry

PBMCs were stimulated according to the previously described method [73] for 3 days using PHA‐L (eBioscience) at 2 μL/mL in a 96‐well plate, which contained FBS‐free X‐VIVO15 medium (Lonza Bioscience). Cells were afterwards treated with 500 ng/mL, 50 μg/mL, 5 μg/mL, 100 μg/mL of farm dust extract or 0.5 ng/mL purified LPS for 24 h. To block nonspecific staining by fluorochrome‐conjugated antibodies, cells were preincubated with Fc Block (BD Bioscience: 10 μg/mL) for 10–15 min on ice. For staining, cells were incubated for 30 min on ice in the dark with antibodies. The following anti‐human surface antigen antibodies were used for Treg cell identification: anti‐CD3‐APC, anti‐CD4‐PE, and anti‐CD25‐Alexa‐flour700. For intracellular staining, cells were fixed and permeabilized using the FOXP3/Transcription Factor Staining Buffer Set (eBioscience) and stained with anti‐FOXP3‐FITC. Frequencies of Treg cells were determined by LSRfortessa II. 200,000 events were acquired and analyzed with FlowJo software (Version 10.10.0, Becton Dickinson, 2019).

### 4.11 Human Treg Cell Stimulation and Cell Phenotyping by Flow Cytometry

PBMCs were stimulated according to the previously described method [73]. Cells were afterward treated with 100 μg/mL farm dust extract once, twice, three, or four times at 24‐h intervals. Cells were analyzed via qPCR (See Table 1) or flow cytometry. The following anti‐human surface antigen antibodies were used for CTLA‐4 in Treg cell identification: anti‐CD4‐APC, anti‐CTLA‐4 PE, and anti‐CD25‐Alexa‐flour700; for intracellular staining, FOXP3/Transcription Factor Staining Buffer Set (eBioscience) was used and stained with anti‐ FOXP3‐FITC. The mean fluorescence intensity of Treg cells was determined by FACSymphony. 200,000 events were acquired per sample and analyzed with FlowJo software (Version 10.10.0, Becton Dickinson, 2019).

**TABLE 1:** List of oligonucleotides.

Target gene	Forward primer (5′–3′)	Reverse primer (5′–3′)
*ß‐Actin*	TGGCACCCAGCACAATGAA	CTAAGTCATAGTCCGCCTAGAAGCA
*Areg*	CCCACACCGTTCACCGAAAT	CTAAGTCATAGTCCGCCTAGAAGCA

### 4.12 Human Th2 Cell Stimulation and Cell Phenotyping by Flow Cytometry

For polarization or stimulation of T helper cells, isolated PBMCs (1 million cells per mL) were stimulated with PMA/Ionomycin (at 50 ng/mL and 1 μg/mL respectively) in the presence of 4 μL BD GolgiStop Protein Transport Inhibitor (BD Bioscience, Cat #554724) per 6 mL of X‐VIVO15 medium (Lonza Bioscience) for 5 h. Cells were harvested and washed two times with PBS. Cells were counted and transferred to the 96‐well plate. Cells were treated with 100 μg/mL farm dust extract once, twice, three times, or four times at 24‐h intervals or four times with 0.5 ng/mL LPS. BD Pharmingen Human Th1/Th2/Th17 Phenotyping Kit was used according to the manufacturer's recommendations for analyzing Th2 cells. Frequencies of Th2 cells were determined by LSRfortessa II or FACSymphony.

### 4.13 Starvation of 16HBE Cells

The 16HBE cell culture was obtained from Millipore Sigma (St. Louis, MO, USA) and used for 15 weeks before returning to frozen cell stocks. After reaching confluence, cells were trypsinized (0.25% trypsin and 2.21 mM EDTA) (Corning Cellgro, Manassas, VA, USA) and then passaged weekly. Cells were seeded at 1.5 × 10^6^ cells per Falcon 75‐cm^2^ culture flask with 25 mL FCS free and Ca^+^ free Dulbecco's Modified Minimum Essential Medium (Corning Cellgro, Manassas, VA, USA). Cultures were incubated at 37°C in 95% air/5% CO_2_ atmosphere for 72 h. Cells were treated with 100 μg/mL farm dust extract once or twice at 24‐h intervals. RNA was extracted from the cells and analyzed via qPCR.

### 4.14 Quantitative Polymerase Chain Reaction

Total RNA was isolated using Nucleo Spin RNA Plus (Macherey Nagel) according to the manufacturer's instructions. Finally, freshly isolated RNAs were reverted into complementary (c)DNAs using the High‐Capacity cDNA Reverse Transcription kit, according to the manufacturer's instructions (Applied Biosystems). Quantitative PCR was done using Syber green (Applied Biosystems) on a QuantStudio 1 Real‐Time PCR System (Thermofisher) using the specific primers (Eurofins, Ebersberg, Germany). As a housekeeping gene, beta‐actin was used [74]. The primers used are described in Table 1.

### 4.15 Statistical Analysis

Statistical analysis for the experimental data was performed using GraphPad Prism version 10 (GraphPad Software Inc., LaJolla, CA, USA). The normal distribution of data was tested using the Kolmogorov–Smirnov and D'Agostino–Pearson tests. When data were normally distributed, statistical differences between the two groups were analyzed by unpaired *t*‐test. If more than two groups were evaluated, the groups were first analyzed by an analysis of variance (one‐way ANOVA), and in case of significance, followed by a Tukey's test. A *p* < 0.05 was considered statistically significant; * represents *p* < 0.05; ** represents *p* < 0.01; *** represents *p* < 0.001; and **** represents *p* < 0.0001.

### 4.16 Single Cell Data Analysis

Processing and statistical analysis of the single cell (sc)RNA‐Seq data was performed using the Seurat V5.0.1 R package [75] and all functions used therein were executed with default parameters—unless otherwise stated. The data was analyzed with the R Core Team (V4.1.3) software. Differential gene expression (DGE) analysis was done using the “FindMarkers” function—with the default parameters. The DimPlot function was used for dimension reduction analysis. A distribution of the absolute (and the percentage) cell counts was computed for all cell types per mouse model—providing a comparative assessment of the abundance of each cell type between the mouse models (Figure 1). The mouse models compared were PBS, OVA, and FDE.

Gene expression profiles were visualized using the DotPlot and Violin functions. Heatmaps for the different gene lists were generated using the “DoHeatmap” function. The function “AverageExpression” was used to compute the average of scaled feature expressions. Data for the immune and non‐immune cells were investigated separately—stratifying by the respective cell types. The data dimensionality reduction approach, uniform manifold approximation, and projection (*UMAP*) was used for the visualization and interpretation of the single‐cell data [76]. The ggplot2 package was used to visualize summary data distributions.

### 4.17 Transepithelial Electrical Resistance (TEER) Measurement

Human primary bronchial epithelial cells were seeded on collagen IV (300 μg/mL) coated 24‐well 0.4 pore diameter PET Transwell membranes (Corning) at a density of 75,000 cells per insert and cultured until confluent. Once the tissue was confluent, differentiation was induced by introducing an air‐liquid interface (ALI) via removal of the apical medium (day 0 of ALI culture), and cells were apically exposed to farm dust extract (100 μg/mL). FDE was repeatedly given starting from d0 of ALI till the end of the experiment (day 35 of ALI): On regular maintenance days (Monday, Wednesday, and Friday of each week), we performed PBS washes on the apical side, followed by fresh application of FDE. For transepithelial electrical resistance (TEER) measurements, 300 μL of media was temporarily added to the apical side of the inserts. Statistical significance and *p*‐value were derived using a paired one‐sided *t*‐test over the mean donor TEER values.

### 4.18 DEG Identification and Enrichment Analysis

Volcano plots were used to visualize differentially expressed genes (DEGs). DEGs for each cell type were subjected to downstream analysis using over‐representation analysis (ORA) and gene‐set enrichment analysis (GSEA) using ClusterProfiler 3.8 [77]. Distinctive marker genes were identified for each cell type based on available DEGs. For each cell type, DEGs were sorted based on |log2(Fold‐Change, FC)| ≥ 0.5 and false discovery rate (FDR) adjusted *p* < 0.05. To gain insight into the underlying molecular mechanisms driving the DEGs, gene ontology (GO) analysis was separately performed for up‐ and down‐regulated genes. Visualization of the functional enrichments following downstream analysis was performed using the R “enrichplot” package. In the heat map from the single‐cell experiments, “higher” and “lower” refer to observed expression trends between conditions and do not reflect the statistical testing.

## Author Contributions

Conceptualization, R.Ü.K., B.R.; methodology, R.Ü.K., J.O.; formal analysis, R.Ü.K., J.O.; investigation, R.Ü.K., J.O., X.T., M.K., G.D., S.C., Z.E., R.A.; resources, C.M.; writing – original draft preparation, R.Ü.K.; writing – review and editing, R.Ü.K., J.O., B.R., S.S., A.Ö.Y., E.v.M., G.D.; visualization, R.Ü.K.; supervision, A.Ö.Y., E.v.M.; project administration, B.R.; funding acquisition, B.R., E.v.M.; design of the experiment, J.N. All authors have read and agreed to the submitted version of the manuscript.

## Conflicts of Interest

Prof. Dr. med. Dr. h.c. Erika von Mutius (E.v.M.) has received research funding from the European Research Council (ERC Advanced Grant), the Bavarian State Ministry of Health and Care (“URS Study”), OM Pharma S.A. (“Impact Chip Study” and “BEAR Study”), and the Federal Ministry of Education and Research (Go Bio Initial Grant, BMBF). E.v.M. is inventor in PCT/US2021/016918, entitled “Therapeutic Fractions and Proteins from Asthma‐Protective Farm Dust.” E.v.M. is inventor in PCT application number EP21189353, entitled “Proteins identified from barn dust extract for the prevention and treatment of diseases.” E.v.M. is inventor in PCT application, serial number PCT/EP2019/085016, entitled “Barn Dust Extract for the Prevention and Treatment of Diseases.” E.v.M. is inventor of the following patents: EP2361632 (“Specific environmental bacteria for the protection from and/or the treatment of allergic, chronic inflammatory and/or autoimmune disorders”), EP1411977 (“Composition containing bacterial antigens used for the prophylaxis and the treatment of allergic diseases”), and EP1637147 (“Stable dust extract for allergy Protection”). E.v.M. received honoraria as an expert from AstraZeneca, HiPP GmbH & Co. KG, OM Pharma S.A., and Böhringer Ingelheim International GmbH. Bettina Rankl (B.R.) is inventor in PCT application number EP21189353, entitled “Proteins identified from barn dust extract for the prevention and treatment of diseases.” The other authors declare no conflicts of interest.

## Supporting information


**Appendix S1:** all70121‐sup‐0001‐AppendixS1.pdf.

**Appendix S2:** all70121‐sup‐0002‐AppendixS2.pptx.

## Data Availability

Data can be provided upon request.

## References

[1] G. Hynes and I. D. Pavord , “Targeted Biologic Therapy for Asthma,” British Medical Bulletin 133 (2020): 16–35, 10.1093/bmb/ldaa004. 32318701

[2] E. Heffler , L. N. G. Madeira , M. Ferrando , et al., “Inhaled Corticosteroids Safety and Adverse Effects in Patients With Asthma,” Journal of Allergy and Clinical Immunology. In Practice 6 (2018): 776–781, 10.1016/j.jaip.2018.01.025. 29408385

[3] I. Axelsson , E. Naumburg , S. O. Prietsch , and L. Zhang , “Inhaled Corticosteroids in Children With Persistent Asthma: Effects of Different Drugs and Delivery Devices on Growth,” Cochrane Database of Systematic Reviews 2019, no. 6 (2019): CD010126, 10.1002/14651858.CD010126.pub2. PMC6564081 31194879

[4] D. W. Cain and J. A. Cidlowski , “Immune Regulation by Glucocorticoids,” Nature Reviews. Immunology 17 (2017): 233–247, 10.1038/nri.2017.1. PMC9761406 28192415

[5] B. E. Chipps , K. R. Murphy , and J. Oppenheimer , “2020 NAEPP Guidelines Update and GINA 2021‐Asthma Care Differences, Overlap, and Challenges,” Journal of Allergy and Clinical Immunology. In Practice 10 (2022): S19–s30, 10.1016/j.jaip.2021.10.032. 34718214

[6] A. L. Buchman , “Side Effects of Corticosteroid Therapy,” Journal of Clinical Gastroenterology 33 (2001): 289–294, 10.1097/00004836-200110000-00006. 11588541

[7] A. Agusti , L. Fabbri , L. Lahousse , D. Singh , and A. Papi , “Single Inhaler Triple Therapy (SITT) in Asthma: Systematic Review and Practice Implications,” Allergy 77 (2022): 1105–1113, 10.1111/all.15076. 34478578 PMC9290056

[8] A. K. Waljee , M. A. Rogers , P. Lin , et al., “Short Term Use of Oral Corticosteroids and Related Harms Among Adults in the United States: Population Based Cohort Study,” BMJ 357 (2017): j1415, 10.1136/bmj.j1415. 28404617 PMC6284230

[9] M. J. Ege , M. Mayer , A. C. Normand , et al., “Exposure to Environmental Microorganisms and Childhood Asthma,” New England Journal of Medicine 364 (2011): 701–709, 10.1056/NEJMoa1007302. 21345099

[10] C. Braun‐Fahrländer , J. Riedler , U. Herz , et al., “Environmental Exposure to Endotoxin and Its Relation to Asthma in School‐Age Children,” New England Journal of Medicine 347 (2002): 869–877, 10.1056/NEJMoa020057. 12239255

[11] M. Peters , “Inhalation of Stable Dust Extract Prevents Allergen Induced Airway Inflammation and Hyperresponsiveness,” Thorax 61 (2006): 134–139, 10.1136/thx.2005.049403. 16244088 PMC2104583

[12] M. M. Stein , C. L. Hrusch , J. Gozdz , et al., “Innate Immunity and Asthma Risk in Amish and Hutterite Farm Children,” New England Journal of Medicine 375 (2016): 411–421, 10.1056/NEJMoa1508749. 27518660 PMC5137793

[13] M. J. Schuijs , M. A. Willart , K. Vergote , et al., “Farm Dust and Endotoxin Protect Against Allergy Through A20 Induction in Lung Epithelial Cells,” Science 349 (2015): 1106–1110, 10.1126/science.aac6623. 26339029

[14] M. Marques Dos Santos , V. Pivniouk , B. Rankl , et al., “Asthma‐Protective Agents in Dust From Traditional Farm Environments,” Journal of Allergy and Clinical Immunology 152 (2023): 610–621, 10.1016/j.jaci.2023.05.013. 37271318 PMC10680491

[15] C. C. Beerweiler , M. Salvermoser , J. Theodorou , et al., “Farm‐Dust Mediated Protection of Childhood Asthma: Mass Cytometry Reveals Novel Cellular Regulation,” Allergy 79 (2024): 3022–3035, 10.1111/all.16347. 39400913

[16] H. Abdala‐Valencia , M. E. Coden , S. E. Chiarella , et al., “Shaping Eosinophil Identity in the Tissue Contexts of Development, Homeostasis, and Disease,” Journal of Leukocyte Biology 104 (2018): 95–108, 10.1002/jlb.1mr1117-442rr. 29656559 PMC6013365

[17] A. V. Wiese , J. Duhn , R. Ü. Korkmaz , et al., “C5aR1 Activation in Mice Controls Inflammatory Eosinophil Recruitment and Functions in Allergic Asthma,” Allergy 78 (2023): 1893–1908, 10.1111/all.15670. 36757006

[18] J. A. O'Sullivan and B. S. Bochner , “Eosinophils and Eosinophil‐Associated Diseases: An Update,” Journal of Allergy and Clinical Immunology 141 (2018): 505–517, 10.1016/j.jaci.2017.09.022. 29045815 PMC5803328

[19] K. Nakagome and M. Nagata , “The Possible Roles of IL‐4/IL‐13 in the Development of Eosinophil‐Predominant Severe Asthma,” Biomolecules 14 (2024): 546, 10.3390/biom14050546. 38785953 PMC11117569

[20] K. D. Stone , C. Prussin , and D. D. Metcalfe , “IgE, Mast Cells, Basophils, and Eosinophils,” Journal of Allergy and Clinical Immunology 125 (2010): S73–S80, 10.1016/j.jaci.2009.11.017. 20176269 PMC2847274

[21] A. Eggel , L. F. Pennington , and T. S. Jardetzky , “Therapeutic Monoclonal Antibodies in Allergy: Targeting IgE, Cytokine, and Alarmin Pathways,” Immunological Reviews 328 (2024): 387–411, 10.1111/imr.13380. 39158477 PMC11659931

[22] W. T. Lee , P. Y. Wu , Y. M. Cheng , and Y. F. Huang , “Tissue Inhibitor of Metalloproteinase 3: Unravelling Its Biological Function and Significance in Oncology,” International Journal of Molecular Sciences 25 (2024): 3191, 10.3390/ijms25063191. 38542164 PMC10970424

[23] C. Beers , A. Burich , M. J. Kleijmeer , J. M. Griffith , P. Wong , and A. Y. Rudensky , “Cathepsin S Controls MHC Class II‐Mediated Antigen Presentation by Epithelial Cells In Vivo,” Journal of Immunology 174 (2005): 1205–1212, 10.4049/jimmunol.174.3.1205. 15661874

[24] C. Klaßen , A. Karabinskaya , L. Dejager , et al., “Airway Epithelial Cells Are Crucial Targets of Glucocorticoids in a Mouse Model of Allergic Asthma,” Journal of Immunology 199 (2017): 48–61, 10.4049/jimmunol.1601691. 28515280

[25] D. M. W. Zaiss , W. C. Gause , L. C. Osborne , and D. Artis , “Emerging Functions of Amphiregulin in Orchestrating Immunity, Inflammation, and Tissue Repair,” Immunity 42 (2015): 216–226, 10.1016/j.immuni.2015.01.020. 25692699 PMC4792035

[26] A. Schmidt , N. Oberle , and P. H. Krammer , “Molecular Mechanisms of Treg‐Mediated T Cell Suppression,” Frontiers in Immunology 3 (2012): 51, 10.3389/fimmu.2012.00051. 22566933 PMC3341960

[27] A. J. Van Oosterhout , C. L. Hofstra , R. Shields , et al., “Murine CTLA4‐IgG Treatment Inhibits Airway Eosinophilia and Hyperresponsiveness and Attenuates IgE Upregulation in a Murine Model of Allergic Asthma,” American Journal of Respiratory Cell and Molecular Biology 17 (1997): 386–392, 10.1165/ajrcmb.17.3.2679. 9308926

[28] X. Zhang , X. Chang Li , X. Xiao , R. Sun , Z. Tian , and H. Wei , “CD4(+)CD62L(+) Central Memory T Cells Can Be Converted to Foxp3(+) T Cells,” PLoS One 8 (2013): e77322, 10.1371/journal.pone.0077322. 24155942 PMC3796486

[29] W. J. Branchett , J. Cook , R. A. Oliver , et al., “Airway Macrophage‐Intrinsic TGF‐β1 Regulates Pulmonary Immunity During Early‐Life Allergen Exposure,” Journal of Allergy and Clinical Immunology 147 (2021): 1892–1906, 10.1016/j.jaci.2021.01.026. 33571538 PMC8098862

[30] N. Sobhani , D. R. Tardiel‐Cyril , A. Davtyan , D. Generali , R. Roudi , and Y. Li , “CTLA‐4 in Regulatory T Cells for Cancer Immunotherapy,” Cancers (Basel) 13 (2021): 1440, 10.3390/cancers13061440. 33809974 PMC8005092

[31] H. Zhao , X. Liao , and Y. Kang , “Tregs: Where We Are and What Comes Next?,” Frontiers in Immunology 8 (2017): 1578, 10.3389/fimmu.2017.01578. 29225597 PMC5705554

[32] J. Ermann , P. Hoffmann , M. Edinger , et al., “Only the CD62L+ Subpopulation of CD4+CD25+ Regulatory T Cells Protects From Lethal Acute GVHD,” Blood 105 (2005): 2220–2226, 10.1182/blood-2004-05-2044. 15546950

[33] R. W. M. Kempkes , I. Joosten , H. Koenen , and X. He , “Metabolic Pathways Involved in Regulatory T Cell Functionality,” Frontiers in Immunology 10 (2019): 2839, 10.3389/fimmu.2019.02839. 31849995 PMC6902900

[34] R. Y. Chen , Y. Zhu , Y. Y. Shen , et al., “The Role of PD‐1 Signaling in Health and Immune‐Related Diseases,” Frontiers in Immunology 14 (2023): 1163633, 10.3389/fimmu.2023.1163633. 37261359 PMC10228652

[35] Y. Han , D. Liu , and L. Li , “PD‐1/PD‐L1 Pathway: Current Researches in Cancer,” American Journal of Cancer Research 10 (2020): 727–742. 32266087 PMC7136921

[36] P. Lao , J. Chen , L. Tang , et al., “Regulatory T Cells in Lung Disease and Transplantation,” Bioscience Reports 43 (2023): 1331, 10.1042/bsr20231331. PMC10611924 37795866

[37] A. J. Thorley , P. A. Ford , M. A. Giembycz , P. Goldstraw , A. Young , and T. D. Tetley , “Differential Regulation of Cytokine Release and Leukocyte Migration by Lipopolysaccharide‐Stimulated Primary Human Lung Alveolar Type II Epithelial Cells and Macrophages,” Journal of Immunology 178 (2007): 463, 10.4049/jimmunol.178.1.463. 17182585

[38] T. H. Lin , C. C. Cheng , H. H. Su , et al., “Lipopolysaccharide Attenuates Induction of Proallergic Cytokines, Thymic Stromal Lymphopoietin, and Interleukin 33 in Respiratory Epithelial Cells Stimulated With PolyI:C and Human Parechovirus,” Frontiers in Immunology 7 (2016): 440, 10.3389/fimmu.2016.00440. 27826297 PMC5078322

[39] C. Cayrol and J. P. Girard , “Interleukin‐33 (IL‐33): A Critical Review of Its Biology and the Mechanisms Involved in Its Release as a Potent Extracellular Cytokine,” Cytokine 156 (2022): 155891, 10.1016/j.cyto.2022.155891. 35640416

[40] S. Wachtendorf , F. Jonin , A. Ochel , et al., “The ST2(+) Treg/Amphiregulin Axis Protects From Immune‐Mediated Hepatitis,” Frontiers in Immunology 15 (2024): 1351405, 10.3389/fimmu.2024.1351405. 38571949 PMC10987816

[41] S. G. Kelsen , I. O. Agache , W. Soong , et al., “Astegolimab (Anti‐ST2) Efficacy and Safety in Adults With Severe Asthma: A Randomized Clinical Trial,” Journal of Allergy and Clinical Immunology 148 (2021): 790–798, 10.1016/j.jaci.2021.03.044. 33872652

[42] C. Schiering , T. Krausgruber , A. Chomka , et al., “The Alarmin IL‐33 Promotes Regulatory T‐Cell Function in the Intestine,” Nature 513 (2014): 564–568, 10.1038/nature13577. 25043027 PMC4339042

[43] D. Préfontaine , S. Lajoie‐Kadoch , S. Foley , et al., “Increased Expression of IL‐33 in Severe Asthma: Evidence of Expression by Airway Smooth Muscle Cells,” Journal of Immunology 183 (2009): 5094–5103, 10.4049/jimmunol.0802387. 19801525

[44] J. Schmitz , A. Owyang , E. Oldham , et al., “IL‐33, an Interleukin‐1‐Like Cytokine That Signals via the IL‐1 Receptor‐Related Protein ST2 and Induces T Helper Type 2‐Associated Cytokines,” Immunity 23 (2005): 479–490, 10.1016/j.immuni.2005.09.015. 16286016

[45] B. S. Mann and K. F. Chung , “Blood Neutrophil Activation Markers in Severe Asthma: Lack of Inhibition by Prednisolone Therapy,” Respiratory Research 7 (2006): 59, 10.1186/1465-9921-7-59. 16600024 PMC1458332

[46] S. H. Kim , U. Uuganbayar , H. K. T. Trinh , et al., “Evaluation of Neutrophil Activation Status According to the Phenotypes of Adult Asthma,” Allergy, Asthma & Immunology Research 11 (2019): 381–393, 10.4168/aair.2019.11.3.381. PMC6439190 30912327

[47] P. L. Bruijnzeel , M. Uddin , and L. Koenderman , “Targeting Neutrophilic Inflammation in Severe Neutrophilic Asthma: Can We Target the Disease‐Relevant Neutrophil Phenotype?,” Journal of Leukocyte Biology 98 (2015): 549–556, 10.1189/jlb.3VMR1214-600RR. 25977288

[48] L. A. Cagle , A. L. Linderholm , L. M. Franzi , et al., “Early Mechanisms of Neutrophil Activation and Transmigration in Acute Lung Injury,” Frontiers in Physiology 13 (2022): 1059686, 10.3389/fphys.2022.1059686. 36620212 PMC9811384

[49] K. F. Chung , “The Role of Airway Smooth Muscle in the Pathogenesis of Airway Wall Remodeling in Chronic Obstructive Pulmonary Disease,” Proceedings of the American Thoracic Society 2 (2005): 347, 10.1513/pats.200504-028SR. 16267361 PMC2713326

[50] R. J. Russell , L. P. Boulet , C. E. Brightling , et al., “The Airway Epithelium: an Orchestrator of Inflammation, a Key Structural Barrier and a Therapeutic Target in Severe Asthma,” European Respiratory Journal 63 (2024): 2301397, 10.1183/13993003.01397-2023. 38453256 PMC10991852

[51] S. F. Rahmawati , R. Vos , I. S. T. Bos , H. A. M. Kerstjens , L. E. M. Kistemaker , and R. Gosens , “Function‐Specific IL‐17A and Dexamethasone Interactions in Primary Human Airway Epithelial Cells,” Scientific Reports 12 (2022): 11110, 10.1038/s41598-022-15393-2. 35773318 PMC9247091

[52] A. J. Giles , M. K. N. D. Hutchinson , H. M. Sonnemann , et al., “Dexamethasone‐Induced Immunosuppression: Mechanisms and Implications for Immunotherapy,” Journal for Immunotherapy of Cancer 6 (2018): 51, 10.1186/s40425-018-0371-5. 29891009 PMC5996496

[53] M. J. Perugorria , M. U. Latasa , A. Nicou , et al., “The Epidermal Growth Factor Receptor Ligand Amphiregulin Participates in the Development of Mouse Liver Fibrosis,” Hepatology 48 (2008): 1251–1261, 10.1002/hep.22437. 18634036

[54] D. M. Zaiss , J. Van Loosdregt , A. Gorlani , et al., “Amphiregulin Enhances Regulatory T Cell‐Suppressive Function via the Epidermal Growth Factor Receptor,” Immunity 38 (2013): 275–284, 10.1016/j.immuni.2012.09.023. 23333074 PMC3582723

[55] Y. Wang and L. Liu , “Immunological Factors, Important Players in the Development of Asthma,” BMC Immunology 25 (2024): 50, 10.1186/s12865-024-00644-w. 39060923 PMC11282818

[56] C. Karagiannidis , M. Akdis , P. Holopainen , et al., “Glucocorticoids Upregulate FOXP3 Expression and Regulatory T Cells in Asthma,” Journal of Allergy and Clinical Immunology 114 (2004): 1425–1433, 10.1016/j.jaci.2004.07.014. 15577848

[57] M. Tekguc , J. B. Wing , M. Osaki , J. Long , and S. Sakaguchi , “Treg‐Expressed CTLA‐4 Depletes CD80/CD86 by Trogocytosis, Releasing Free PD‐L1 on Antigen‐Presenting Cells,” Proceedings of the National Academy of Sciences of the United States of America 118, no. 30 e2023739118 (2021), 10.1073/pnas.2023739118. 34301886 PMC8325248

[58] D. G. Helou , C. Quach , M. Fung , et al., “Human PD‐1 Agonist Treatment Alleviates Neutrophilic Asthma by Reprogramming T Cells,” Journal of Allergy and Clinical Immunology 151 (2023): 526–538.e528, 10.1016/j.jaci.2022.07.022. 35963455 PMC9905221

[59] C. L. Hrusch , M. M. Stein , J. Gozdz , et al., “T‐Cell Phenotypes Are Associated With Serum IgE Levels in Amish and Hutterite Children,” Journal of Allergy and Clinical Immunology 144 (2019): 1391–1401.e1310, 10.1016/j.jaci.2019.07.034. 31401285 PMC6842432

[60] S. Navarro , G. Cossalter , C. Chiavaroli , et al., “The Oral Administration of Bacterial Extracts Prevents Asthma via the Recruitment of Regulatory T Cells to the Airways,” Mucosal Immunology 4 (2011): 53–65, 10.1038/mi.2010.51. 20811345

[61] V. Pivniouk , J. A. Gimenes‐Junior , P. Ezeh , et al., “Airway Administration of OM‐85, a Bacterial Lysate, Blocks Experimental Asthma by Targeting Dendritic Cells and the Epithelium/IL‐33/ILC2 Axis,” Journal of Allergy and Clinical Immunology 149 (2022): 943–956, 10.1016/j.jaci.2021.09.013. 34560105 PMC8901455

[62] H. Chanteux , A. C. Guisset , C. Pilette , and Y. Sibille , “LPS Induces IL‐10 Production by Human Alveolar Macrophages via MAPKinases‐ and Sp1‐Dependent Mechanisms,” Respiratory Research 8 (2007): 71, 10.1186/1465-9921-8-71. 17916230 PMC2080632

[63] D. V. Chistyakov , N. V. Azbukina , A. A. Astakhova , S. V. Goriainov , V. V. Chistyakov , and M. G. Sergeeva , “Sex‐Mediated Differences in LPS Induced Alterations of TNFα, IL‐10 Expression, and Prostaglandin Synthesis in Primary Astrocytes,” International Journal of Molecular Sciences 19 (2018): 2793, 10.3390/ijms19092793. 30227622 PMC6164227

[64] X. Liu , M. Li , Y. Wu , Y. Zhou , L. Zeng , and T. Huang , “Anti‐IL‐33 Antibody Treatment Inhibits Airway Inflammation in a Murine Model of Allergic Asthma,” Biochemical and Biophysical Research Communications 386 (2009): 181–185, 10.1016/j.bbrc.2009.06.008. 19508862

[65] F. R. Shardonofsky , J. Venzor , R. Barrios , K. P. Leong , and D. P. Huston , “Therapeutic Efficacy of an Anti‐IL‐5 Monoclonal Antibody Delivered Into the Respiratory Tract in a Murine Model of Asthma,” Journal of Allergy and Clinical Immunology 104 (1999): 215–221, 10.1016/s0091-6749(99)70138-7. 10400864

[66] M. X. Xia , X. Ding , J. Qi , J. Gu , G. Hu , and X. L. Sun , “Inhaled Budesonide Protects Against Chronic Asthma‐Induced Neuroinflammation in Mouse Brain,” Journal of Neuroimmunology 273 (2014): 53–57, 10.1016/j.jneuroim.2014.06.005. 24993070

[67] C. C. Lin , K. C. Chuang , S. W. Chen , et al., “Lactoferrin Ameliorates Ovalbumin‐Induced Asthma in Mice Through Reducing Dendritic‐Cell‐Derived Th2 Cell Responses,” International Journal of Molecular Sciences 23 (2022): 14185, 10.3390/ijms232214185. 36430662 PMC9696322

[68] C. Vock , A. Ö. Yildirim , C. Wagner , et al., “Distal Airways Are Protected From Goblet Cell Metaplasia by Diminished Expression of IL‐13 Signalling Components,” Clinical and Experimental Allergy 45 (2015): 1447–1458, 10.1111/cea.12526. 25772331

[69] K. Antoniou , F. Ender , T. Vollbrandt , et al., “Allergen‐Induced C5a/C5aR1 Axis Activation in Pulmonary CD11b(+) cDCs Promotes Pulmonary Tolerance Through Downregulation of CD40,” Cells 9 (2020): 300, 10.3390/cells9020300. 31991941 PMC7072238

[70] I. Schmudde , H. A. Ströver , T. Vollbrandt , et al., “C5a Receptor Signalling in Dendritic Cells Controls the Development of Maladaptive Th2 and Th17 Immunity in Experimental Allergic Asthma,” Mucosal Immunology 6 (2013): 807–825, 10.1038/mi.2012.119. 23212198

[71] A. V. Wiese , F. Ender , K. M. Quell , et al., “The C5a/C5aR1 Axis Controls the Development of Experimental Allergic Asthma Independent of LysM‐Expressing Pulmonary Immune Cells,” PLoS One 12 (2017): e0184956, 10.1371/journal.pone.0184956. 28931049 PMC5607179

[72] F. Ender , A. V. Wiese , I. Schmudde , et al., “Differential Regulation of C5a Receptor 1 in Innate Immune Cells During the Allergic Asthma Effector Phase,” PLoS One 12 (2017): e0172446, 10.1371/journal.pone.0172446. 28231307 PMC5322932

[73] J. Jiao , X. Zhao , R. Hou , et al., “Comparison of Two Commonly Used Methods for Stimulating T Cells,” Biotechnology Letters 41 (2019): 1361–1371, 10.1007/s10529-019-02743-w. 31631231

[74] T. Makino , M. Mizawa , K. Takemoto , S. Yamamoto , and T. Shimizu , “Effect of Tumour Necrotic Factor‐α, Interleukin‐17 and Interleukin‐22 on the Expression of Filaggerin‐2 and Hornerin: Analysis of a Three‐Dimensional Psoriatic Skin Model,” Skin Health and Disease 4 (2024): e440, 10.1002/ski2.440. 39624730 PMC11608895

[75] Y. Hao , T. Stuart , M. H. Kowalski , et al., “Dictionary Learning for Integrative, Multimodal and Scalable Single‐Cell Analysis,” Nature Biotechnology 42 (2024): 293–304, 10.1038/s41587-023-01767-y. PMC10928517 37231261

[76] E. Becht , L. McInnes , J. Healy , et al., “Dimensionality Reduction for Visualizing Single‐Cell Data Using UMAP,” Nature Biotechnology 37 (2018): 38–44, 10.1038/nbt.4314. 30531897

[77] G. Yu , L. G. Wang , Y. Han , and Q. Y. He , “clusterProfiler: An R Package for Comparing Biological Themes Among Gene Clusters,” OMICS 16 (2012): 284–287, 10.1089/omi.2011.0118. 22455463 PMC3339379

