# Supplementary material for: The Therapeutic Potential of Farm Dust Extracts in a Mouse Model of Eosinophilic Inflammation
Source: Allergy. 2025 Oct 22;81(4):1173–92. doi: 10.1111/all.70121 (PMC13040664; doi:10.1111/all.70121)
Supplement: Supplementary file 1 — Appendix S1: all70121‐sup‐0001‐AppendixS1.pdf. [file ALL-81-1173-s001.pdf]

Supplementary information

Figure S1. (**A**) Compliance of the respiratory system (Crs) was determined after i.n., PBS- (blue line), OVA-challenged (red line), and FDE treatment (purple line). Crs was measured as airway resistance in response to methacholine exposure. Data are presented as mean ± SEM. n = 3 mice per group. Statistical significance was determined by one-way ANOVA with Tukey's post hoc test; *p < 0.05, **p < 0.01, ***p < 0.001. (**B**) Elastance of the respiratory system (ERS), determined after i.n. PBS- (blue line), OVA-challenged (red line), and FDE treatment (purple line). Data are presented as mean ± SEM. n = 3 mice per group. Statistical significance was determined by one-way ANOVA with Tukey's post hoc test. (**C**) Gating strategy to identify pulmonary, CD103+ cDCs, CD11b+ cDCs and moDCs in BALB/c mice. CD11b+ cDCs were identified as SiglecF− lin− CD11C+ MHC-IIhi CD103− CD11b+ CD64− cells. moDCs were identified as SiglecF− lin− CD11C+ MHC-IIhi CD103− CD11b+ CD64+ cells and CD103+ cDCs were identified as SiglecF− lin− CD11C+ MHC-IIhi CD103+ cells. (**D**) MFI of CD103 on DC, PBS- (solid Turquoise), OVA-challenged (solid red), and FDE treated (solid purple), or i.p. dexamethasone treatment (solid blue). (**E**) Fluorescence-minus-one (FMO) controls for Foxp3 and CD25 in Treg cells. The gating strategy included pre-gating on $CD4^{+}CD3^{+}$ cells to accurately identify the regulatory T cell population. (**F**) Gating strategy to identify cells in BALF, Contour plots of lung cells from PBS (upper left panel), OVA (lower right panel), FDE (upper right panel), and Dexamethasone (lower left panel) treated mice. Eosinophils (Eos) were identified as SiglecF+ CD11C-, alveolar macrophages (AMs) were identified as SiglecF+ CD11C+, neutrophils were identified as SiglecF− Ly6g+, T cells were identified as SiglecF− CD4+ cells.

Figure S2. BALF level of IL-17A, IL2, and TNF-α and serum level of IL-22, IL-17a, TNF-α, and IFN-ɣ. PBS (upper left panel), OVA (lower right panel), FDE (upper right panel), and Dexamethasone (lower left panel) treated mice. Data shown are the mean ± SEM; n = 3-6. Data were analyzed by ANOVA followed by a Tukey test.

Figure S3. (**A**) Number of cells in the epithelial compartment analyzed by single-cell RNA sequencing, comparing FDE- and PBS-treated lungs. (**B**) Percentage of cells within the non-epithelial compartment after FDE treatment, based on single-cell analysis. (**C**) Number of cells in the immune cell compartment analyzed by single-cell RNA sequencing, comparing FDE- and PBS-treated lungs. (**D**) Percentage of cells within the immune compartment after FDE treatment, based on single-cell analysis. (**E**) Predicted cell-cell communication analysis among all immune cells, illustrating the interaction networks identified by single-cell analysis.(**F**) Treg cell-specific, cell-cell communication patterns. Abbreviations: Monocyte-derived macrophages (MDM), Cytotoxic T cells (CTL), Cycling macrophages (CyM), Immature neutrophils (ImNs), Lung-resident macrophages, (LRM)Mast cells/Basophils (MCB)

Figure S4. (**A**) Heat map analysis in Neutrophil cells based on key surface marker expression profiles. (**B**) Heat map analysis in AT2 cells based on gene expression profiles of key MHC-II genes. (**C**) Heat map analysis in goblet cells based on key gene expression profiles associated with mucus formation. (**D**)Heat map analysis in club cells based on key gene expression profiles associated with mucus formation. (**E**) Heat map analysis in T cells based on gene expression profiles of key MHC-II genes (**F**)Monocyte-derived macrophages (MDM)s based on gene expression profiles of key MHC-II genes. (**G**) Quantification of AMs numbers upon PBS- (white bar), OVA-challenged (red bar), and FDE (purple bar) or Dexamethasone-treated mice (blue bar). Data were analyzed by ANOVA followed by a Tukey test; Data show ΔMFI ± SEM; n = 3-6. (**H**) Quantification of IMs numbers upon PBS- (white bar), OVA-challenged (red bar), and FDE (purple bar) or Dexamethasone-treated mice (blue bar). Data were analyzed by ANOVA followed by a Tukey test; Data show ΔMFI ± SEM; n = 3-6.

Figure S5. (**A**) Frequency of PAS-positive cells (PBS- white bar), OVA-challenged (red bar), and FDE (purple bar), or i.p. dexamethasone treatment (blue bar). Data were analyzed by ANOVA followed by Tukey's post hoc test. (**B**) Airway inflammation by H&E scoring (PBS- white bar), OVA-challenged (red bar), FDE (purple bar), or i.p. dexamethasone treatment (blue bar). Data were analyzed by ANOVA followed by Tukey's post hoc test.

Figure S6. Assessment of fibrosis- and COPD-related gene expression following FDE treatment using single-cell RNA sequencing.(A) Gene expression profiles of canonical COPD markers across immune (A) and epithelial (B) compartments.(C–D) Expression of key fibrosis-associated genes in immune (C) and epithelial (D) cell populations.

Figure S7. Heat map analysis in macrophages based on genes associated with the canonical TGF-β signaling pathway. Group 1, which includes genes that previously have been strongly associated with severe wheezing and chronic cough in human populations, and Group 2, comprises genes less associated with these symptoms.

Figure S1

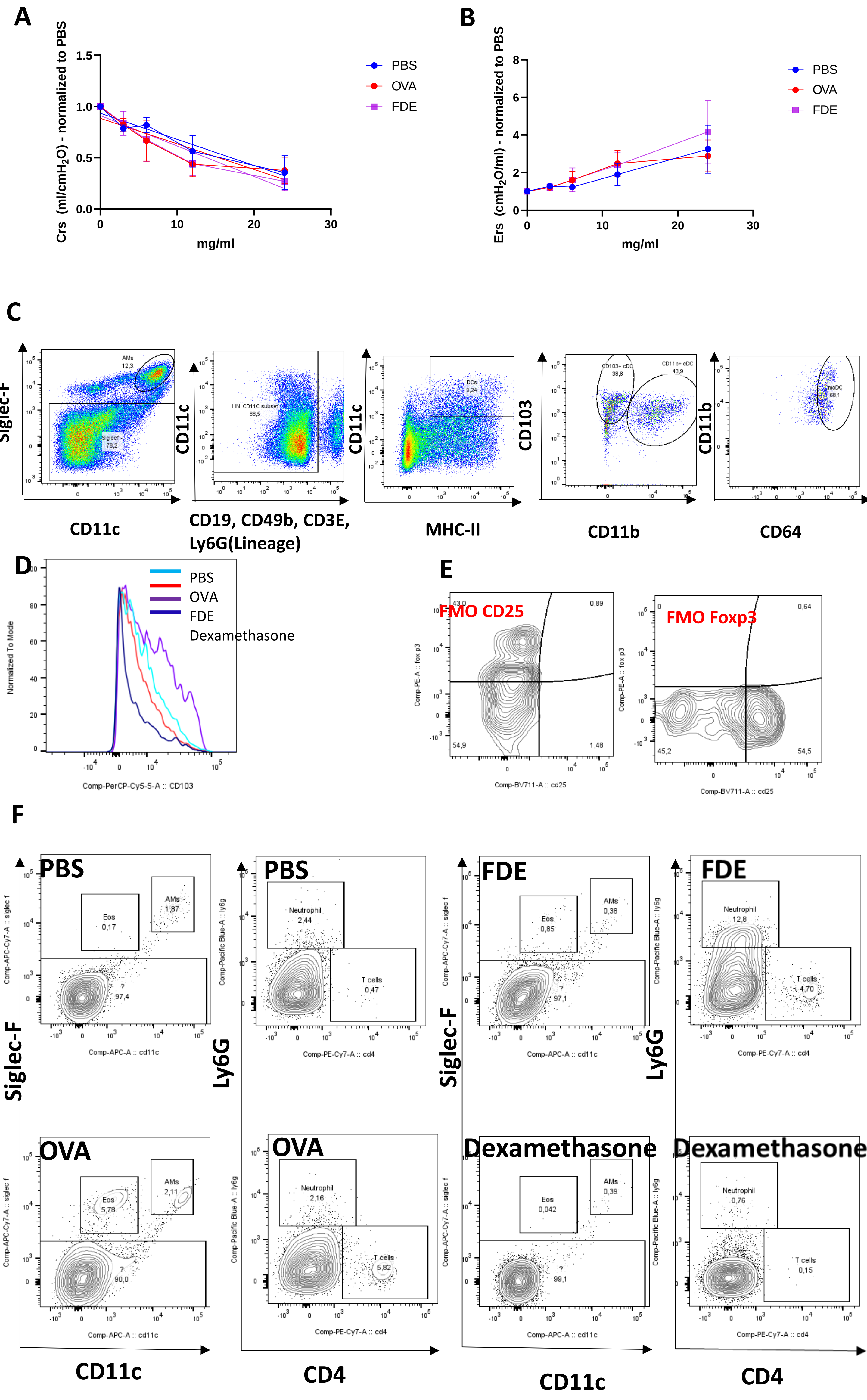

A
Crs (ml/cmH2O) - normalized to PBS
mg/ml
PBS
OVA
FDE
B
Ers (cmH2O/ml) - normalized to PBS
mg/ml
PBS
OVA
FDE
C
Siglec-F
CD11c
CD19, CD49b, CD3E, Ly6G(Lineage)
MHC-II
CD103
CD11b
CD64
D
PBS
OVA
FDE
Dexamethasone
Normalized To Mode
Comp-PerCP-Cy5-5-A :: CD103
E
FMO CD25
FMO Foxp3
Comp-PE-A :: fox p3
Comp-BV711-A :: cd25
F
PBS
OVA
FDE
Dexamethasone
Siglec-F
Ly6G
CD11c
CD4
Comp-APC-Cy7-A :: siglec f
Comp-APC-A :: cd11c
Comp-Pacific Blue-A :: ly6g
Comp-PE-Cy7-A :: cd4

Figure S2

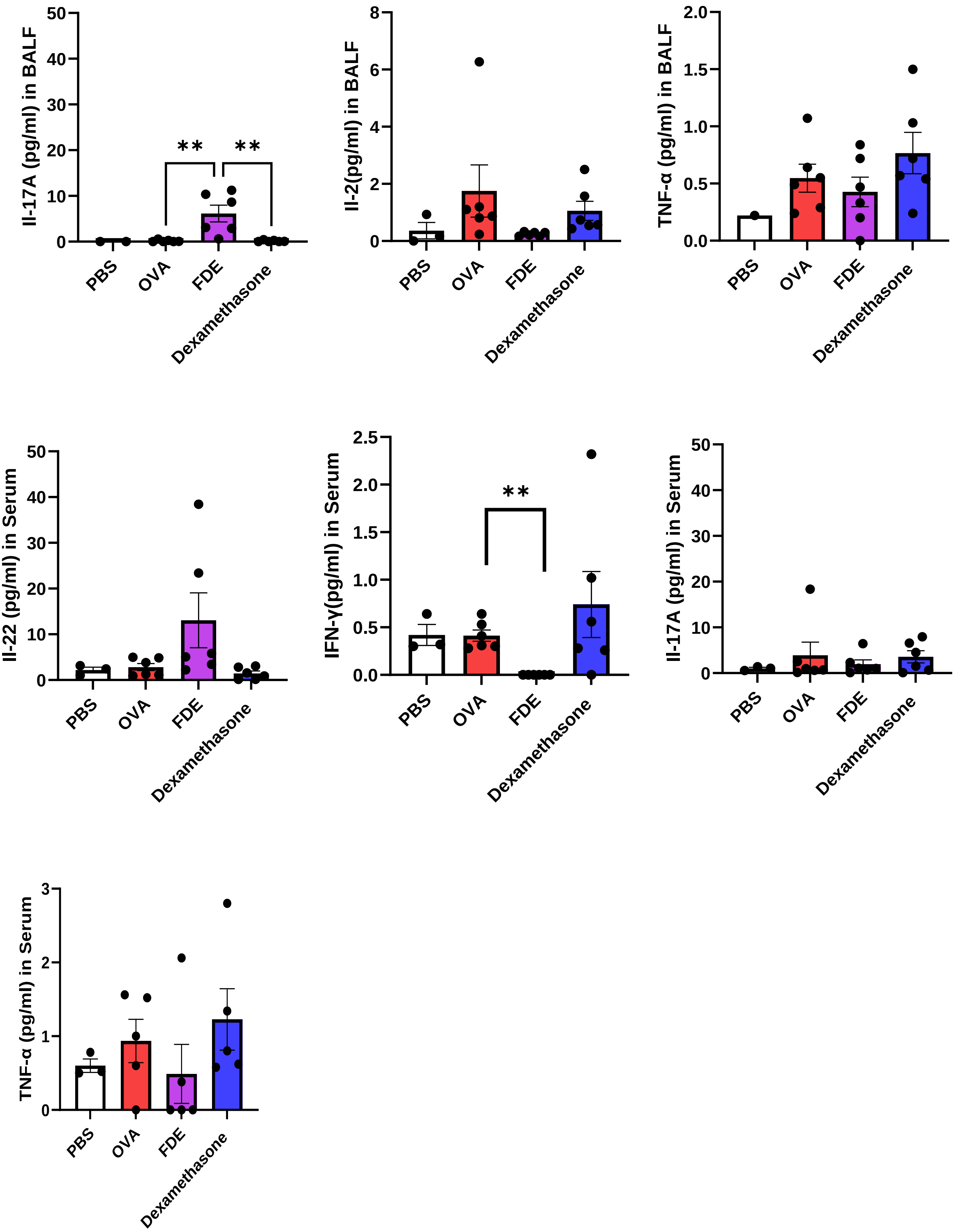
Il-17A (pg/ml) in BALF
Il-2(pg/ml) in BALF
TNF-α (pg/ml) in BALF
Il-22 (pg/ml) in Serum
IFN-γ(pg/ml) in Serum
Il-17A (pg/ml) in Serum
TNF-α (pg/ml) in Serum
PBS
OVA
FDE
Dexamethasone
**
**
**

Figure S3

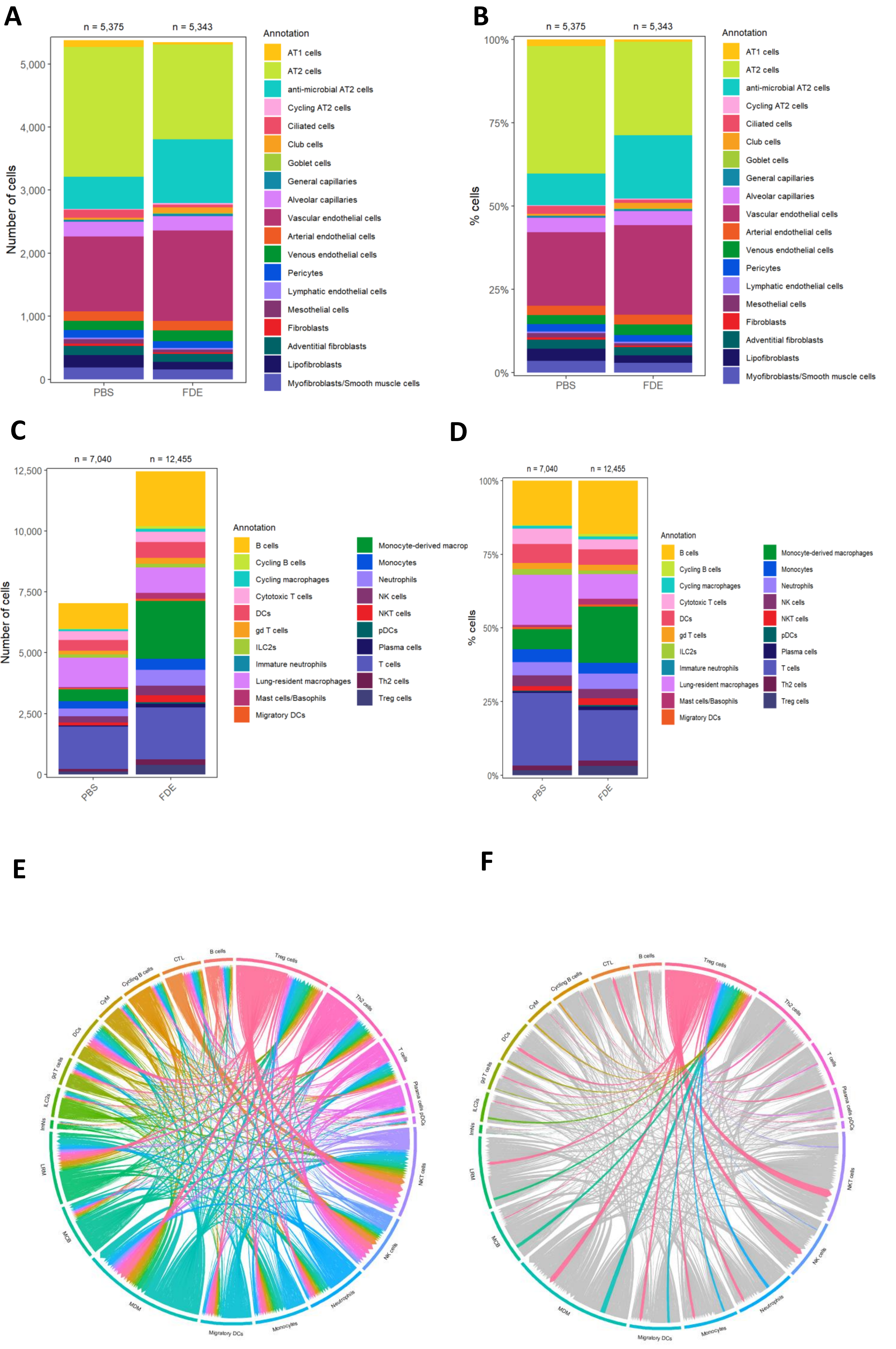

A
n = 5,375
n = 5,343
Number of cells
5,000
4,000
3,000
2,000
1,000
0
PBS
FDE
Annotation
AT1 cells
AT2 cells
anti-microbial AT2 cells
Cycling AT2 cells
Ciliated cells
Club cells
Goblet cells
General capillaries
Alveolar capillaries
Vascular endothelial cells
Arterial endothelial cells
Venous endothelial cells
Pericytes
Lymphatic endothelial cells
Mesothelial cells
Fibroblasts
Adventitial fibroblasts
Lipofibroblasts
Myofibroblasts/Smooth muscle cells
B
n = 5,375
n = 5,343
% cells
100%
75%
50%
25%
0%
PBS
FDE
Annotation
AT1 cells
AT2 cells
anti-microbial AT2 cells
Cycling AT2 cells
Ciliated cells
Club cells
Goblet cells
General capillaries
Alveolar capillaries
Vascular endothelial cells
Arterial endothelial cells
Venous endothelial cells
Pericytes
Lymphatic endothelial cells
Mesothelial cells
Fibroblasts
Adventitial fibroblasts
Lipofibroblasts
Myofibroblasts/Smooth muscle cells
C
n = 7,040
n = 12,455
Number of cells
12,500
10,000
7,500
5,000
2,500
0
PBS
FDE
Annotation
B cells
Cycling B cells
Cycling macrophages
Cytotoxic T cells
DCs
gd T cells
ILC2s
Immature neutrophils
Lung-resident macrophages
Mast cells/Basophils
Migratory DCs
Monocyte-derived macrop
Monocytes
Neutrophils
NK cells
NKT cells
pDCs
Plasma cells
T cells
Th2 cells
Treg cells
D
n = 7,040
n = 12,455
% cells
100%
75%
50%
25%
0%
PBS
FDE
Annotation
B cells
Cycling B cells
Cycling macrophages
Cytotoxic T cells
DCs
gd T cells
ILC2s
Immature neutrophils
Lung-resident macrophages
Mast cells/Basophils
Migratory DCs
Monocyte-derived macrophages
Monocytes
Neutrophils
NK cells
NKT cells
pDCs
Plasma cells
T cells
Th2 cells
Treg cells
E
B cells
Treg cells
Th2 cells
T cells
Plasma cells pDCs
NKT cells
NK cells
Neutrophils
Monocytes
Migratory DCs
MDM
MCB
LRM
ImNs
ILC2s
gd T cells
DCs
CyM
Cycling B cells
CTL
F
B cells
Treg cells
Th2 cells
T cells
Plasma cells pDCs
NKT cells
NK cells
Neutrophils
Monocytes
Migratory DCs
MDM
MCB
LRM
ImNs
ILC2s
gd T cells
DCs
CyM
Cycling B cells
CTL

Figure S4

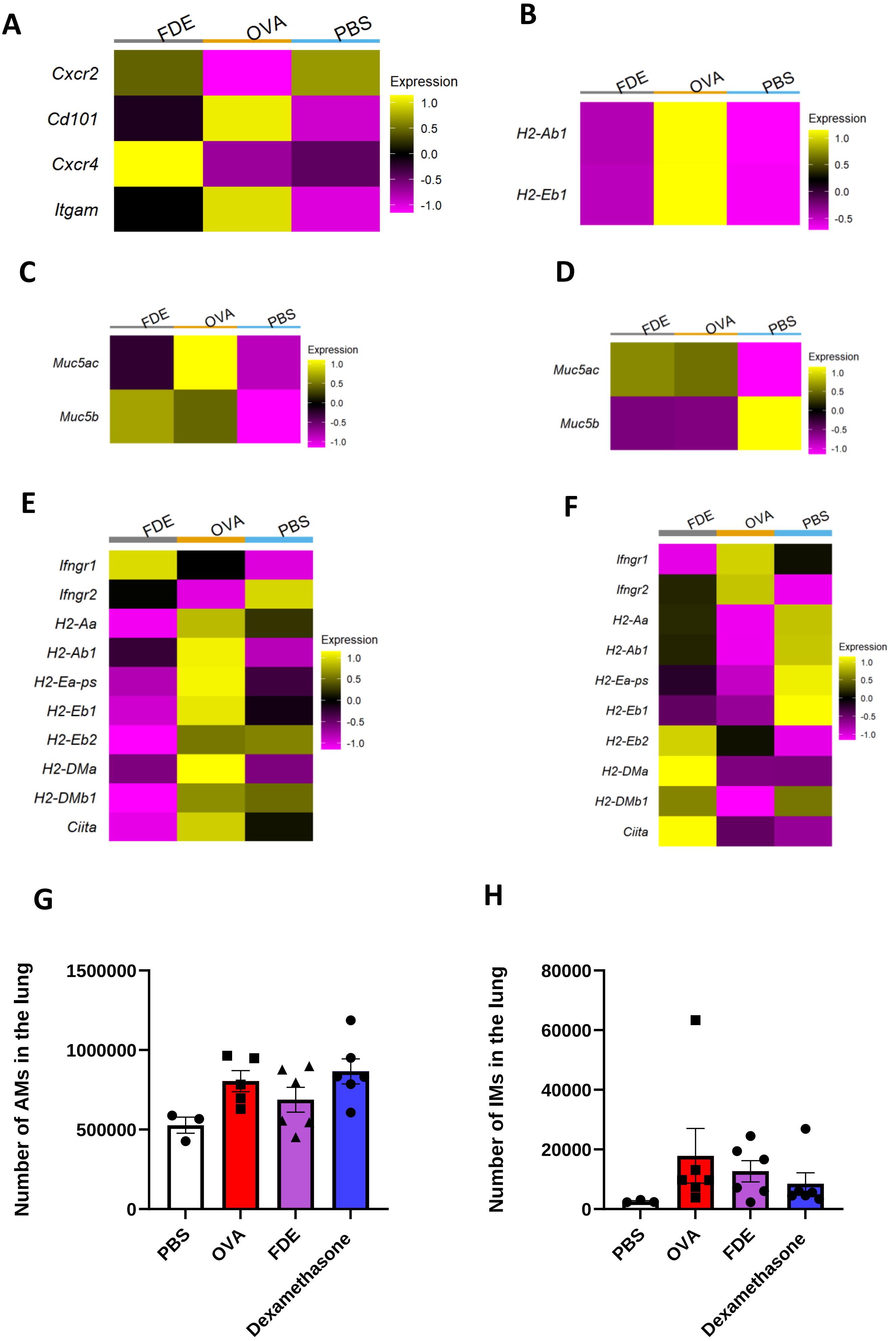

A
FDE
OVA
PBS
Cxcr2
Cd101
Cxcr4
Itgam
Expression
1.0
0.5
0.0
-0.5
-1.0
B
FDE
OVA
PBS
H2-Ab1
H2-Eb1
Expression
1.0
0.5
0.0
-0.5
C
FDE
OVA
PBS
Muc5ac
Muc5b
Expression
1.0
0.5
0.0
-0.5
-1.0
D
FDE
OVA
PBS
Muc5ac
Muc5b
Expression
1.0
0.5
0.0
-0.5
-1.0
E
FDE
OVA
PBS
Ifngr1
Ifngr2
H2-Aa
H2-Ab1
H2-Ea-ps
H2-Eb1
H2-Eb2
H2-DMa
H2-DMb1
Ciita
Expression
1.0
0.5
0.0
-0.5
-1.0
F
FDE
OVA
PBS
Ifngr1
Ifngr2
H2-Aa
H2-Ab1
H2-Ea-ps
H2-Eb1
H2-Eb2
H2-DMa
H2-DMb1
Ciita
Expression
1.0
0.5
0.0
-0.5
-1.0
G
Number of AMs in the lung
1500000
1000000
500000
0
PBS
OVA
FDE
Dexamethasone
H
Number of IMs in the lung
80000
60000
40000
20000
0
PBS
OVA
FDE
Dexamethasone

Figure S5

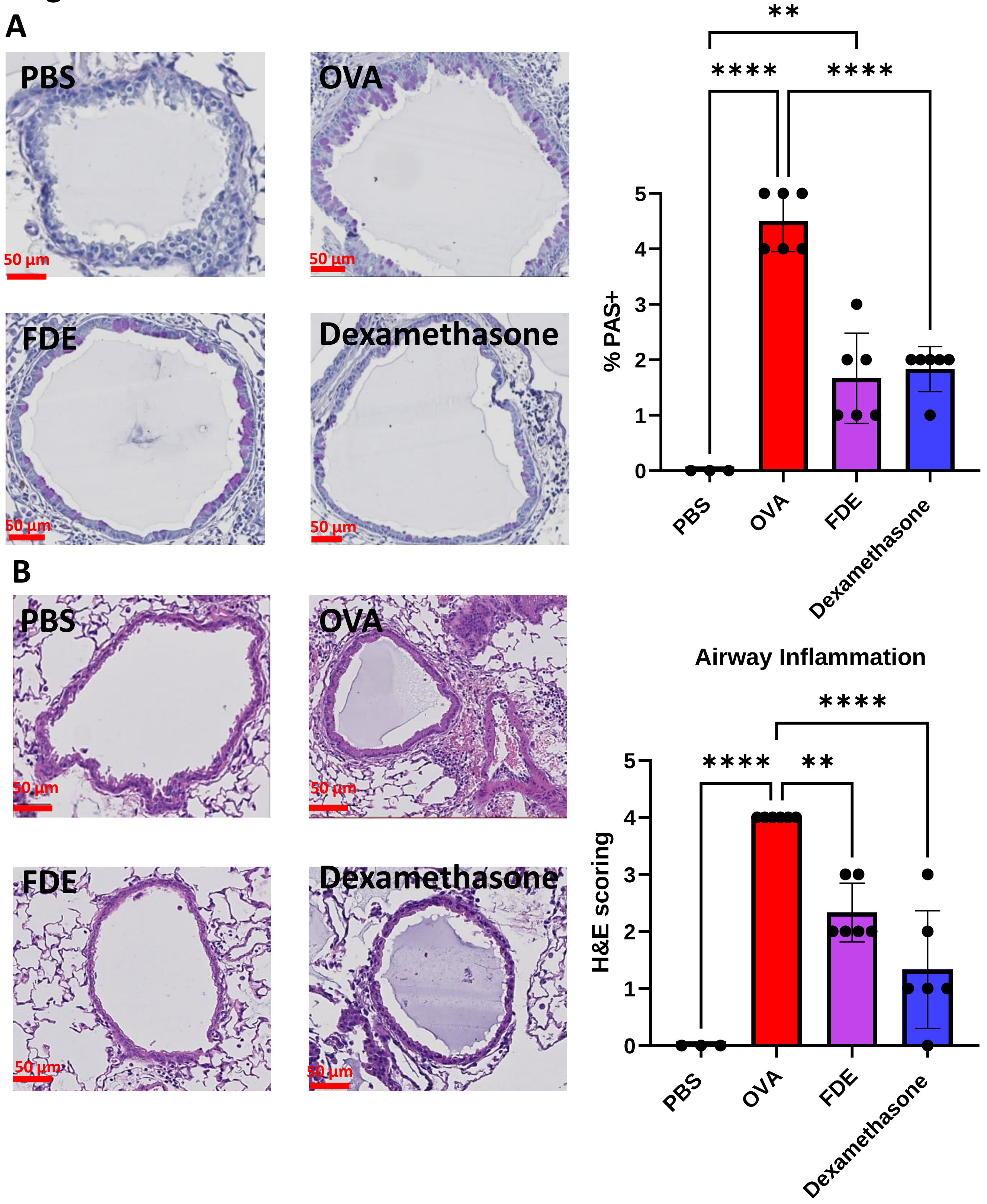
A
PBS
OVA
FDE
Dexamethasone
50 µm
% PAS+
0
1
2
3
4
5
**
****
****
PBS
OVA
FDE
Dexamethasone
B
PBS
OVA
FDE
Dexamethasone
50 µm
Airway Inflammation
H&E scoring
0
1
2
3
4
5
****
****
**
PBS
OVA
FDE
Dexamethasone

Figure S6

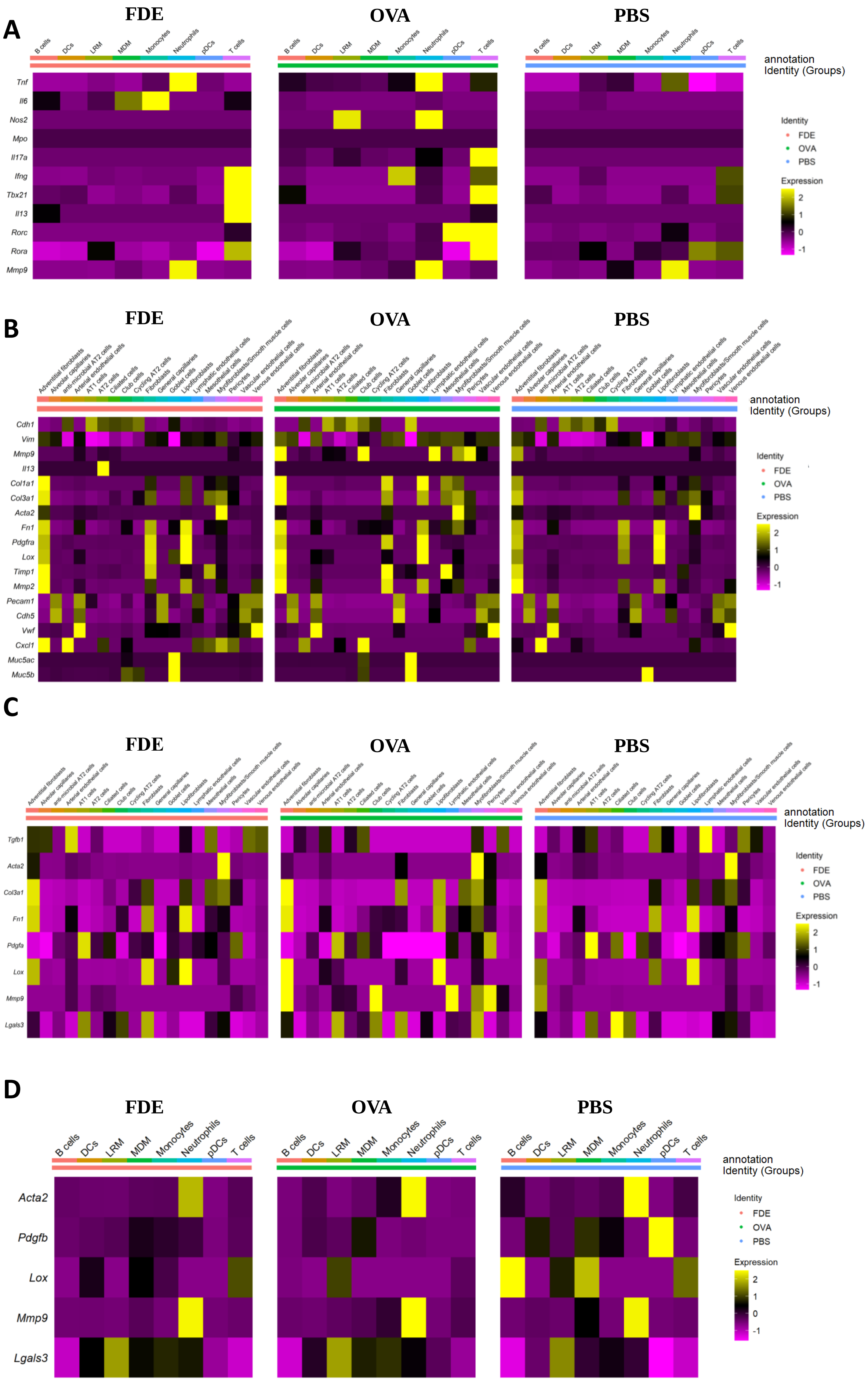
A
B
C
D
FDE
OVA
PBS
B cells
DCs
LRM
MDM
Monocytes
Neutrophils
pDCs
T cells
Tnf
Il6
Nos2
Mpo
Il17a
Ifng
Tbx21
Il13
Rorc
Rora
Mmp9
Adventitial fibroblasts
Alveolar capillaries
anti-microbial AT2 cells
Arterial endothelial cells
AT1 cells
AT2 cells
Ciliated cells
Club cells
Cycling AT2 cells
Fibroblasts
General capillaries
Goblet cells
Lipofibroblasts
Lymphatic endothelial cells
Mesothelial cells
Myofibroblasts/Smooth muscle cells
Pericytes
Vascular endothelial cells
Venous endothelial cells
Cdh1
Vim
Mmp9
Il13
Col1a1
Col3a1
Acta2
Fn1
Pdgfra
Lox
Timp1
Mmp2
Pecam1
Cdh5
Vwf
Cxcl1
Muc5ac
Muc5b
Tgfb1
Acta2
Col3a1
Fn1
Pdgfa
Lox
Mmp9
Lgals3
Acta2
Pdgfb
Lox
Mmp9
Lgals3
annotation
Identity (Groups)
Identity
FDE
OVA
PBS
Expression
2
1
0
-1

Figure S7

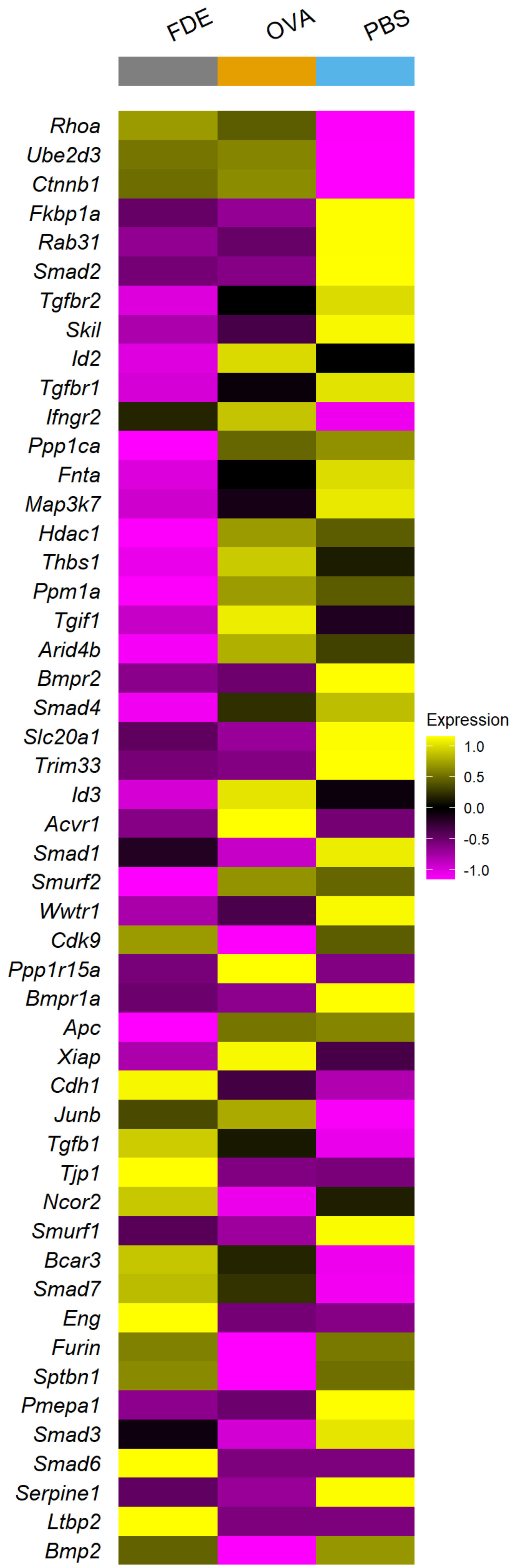
FDE
OVA
PBS
Rhoa
Ube2d3
Ctnnb1
Fkbp1a
Rab31
Smad2
Tgfbr2
Skil
Id2
Tgfbr1
Ifngr2
Ppp1ca
Fnta
Map3k7
Hdac1
Thbs1
Ppm1a
Tgif1
Arid4b
Bmpr2
Smad4
Slc20a1
Trim33
Id3
Acvr1
Smad1
Smurf2
Wwtr1
Cdk9
Ppp1r15a
Bmpr1a
Apc
Xiap
Cdh1
Junb
Tgfb1
Tjp1
Ncor2
Smurf1
Bcar3
Smad7
Eng
Furin
Sptbn1
Pmepa1
Smad3
Smad6
Serpine1
Ltbp2
Bmp2
Expression
1.0
0.5
0.0
-0.5
-1.0
