# Supplementary material for: The Therapeutic Potential of Farm Dust Extracts in a Mouse Model of Eosinophilic Inflammation
Source: Allergy. 2025 Oct 22;81(4):1173–92. doi: 10.1111/all.70121 (PMC13040664; doi:10.1111/all.70121)
Supplement: Supplementary file 2 — Appendix S2: all70121‐sup‐0002‐AppendixS2.pptx. [file ALL-81-1173-s002.pptx]

## Slide 1
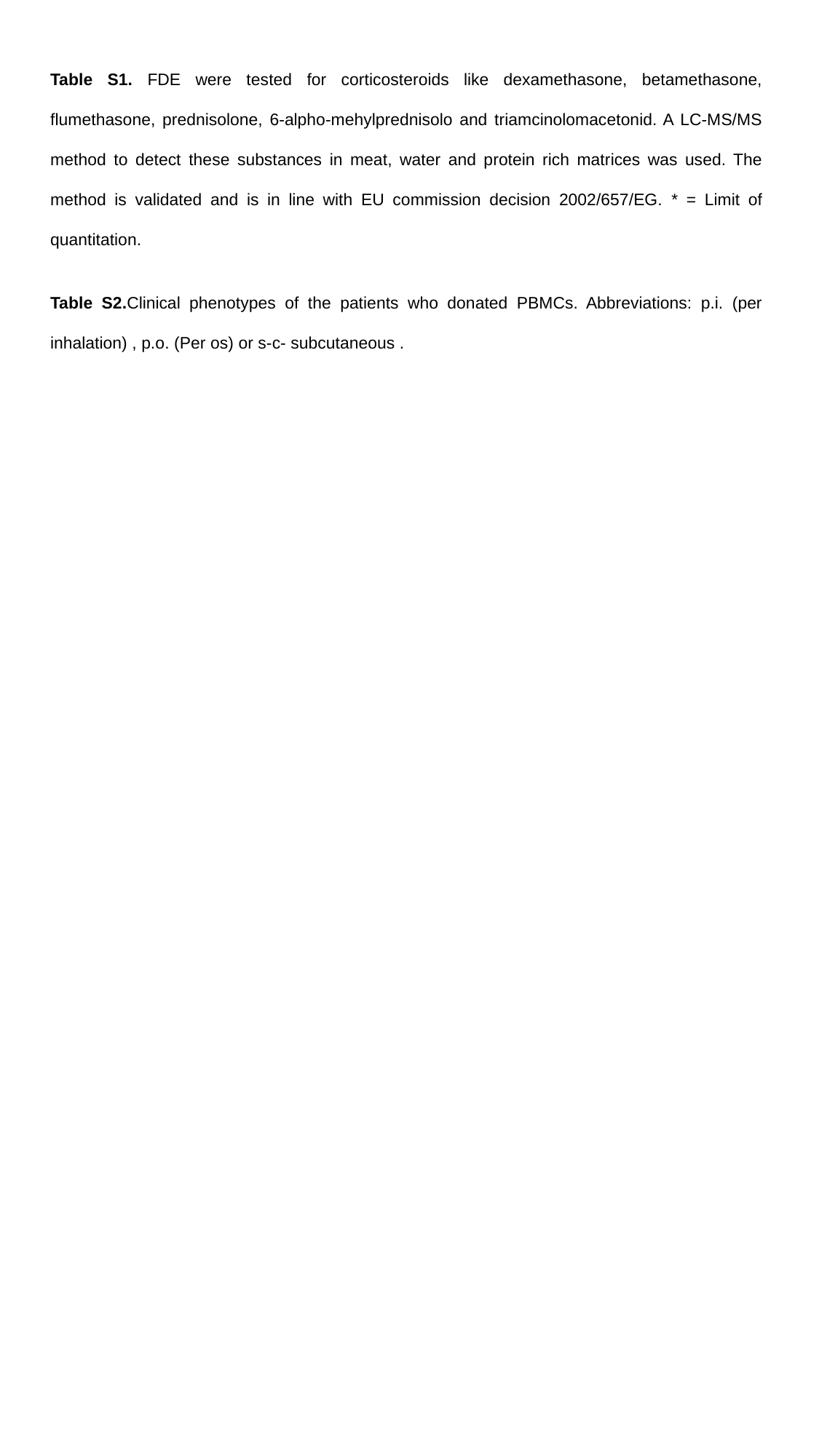

Table S1. FDE were tested for corticosteroids like dexamethasone, betamethasone, flumethasone, prednisolone, 6-alpho-mehylprednisolo and triamcinolomacetonid. A LC-MS/MS method to detect these substances in meat, water and protein rich matrices was used. The method is validated and is in line with EU commission decision 2002/657/EG. * = Limit of quantitation.
Table S2.Clinical phenotypes of the patients who donated PBMCs. Abbreviations: p.i. (per inhalation) , p.o. (Per os) or s-c- subcutaneous .

## Slide 2
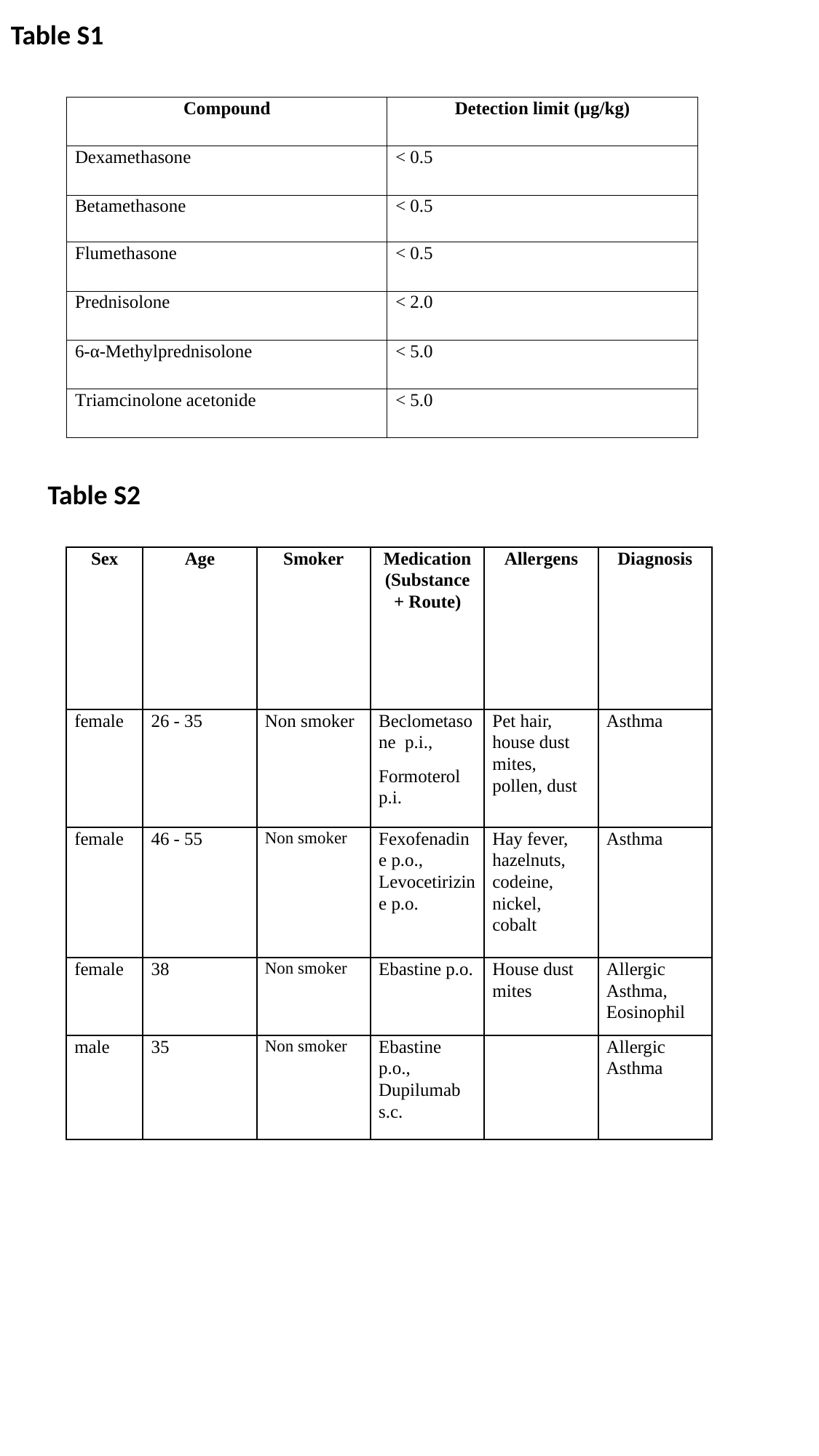

Table S1
Table S2
| Sex | Age | Smoker | Medication (Substance + Route) | Allergens | Diagnosis |
| --- | --- | --- | --- | --- | --- |
| female | 26 - 35 | Non smoker | Beclometasone p.i., Formoterol p.i. | Pet hair, house dust mites, pollen, dust | Asthma |
| female | 46 - 55 | Non smoker | Fexofenadine p.o., Levocetirizine p.o. | Hay fever, hazelnuts, codeine, nickel, cobalt | Asthma |
| female | 38 | Non smoker | Ebastine p.o. | House dust mites | Allergic Asthma, Eosinophil |
| male | 35 | Non smoker | Ebastine p.o., Dupilumab s.c. | | Allergic Asthma |
